# Observing Aqueous Proton-Uptake Reactions Triggered by Light

**DOI:** 10.1021/jacs.2c11441

**Published:** 2023-03-20

**Authors:** Balázs Antalicz, Jan Versluis, Huib J. Bakker

**Affiliations:** AMOLF, Ultrafast Spectroscopy, Science Park 104, 1098 XG Amsterdam, The Netherlands

## Abstract

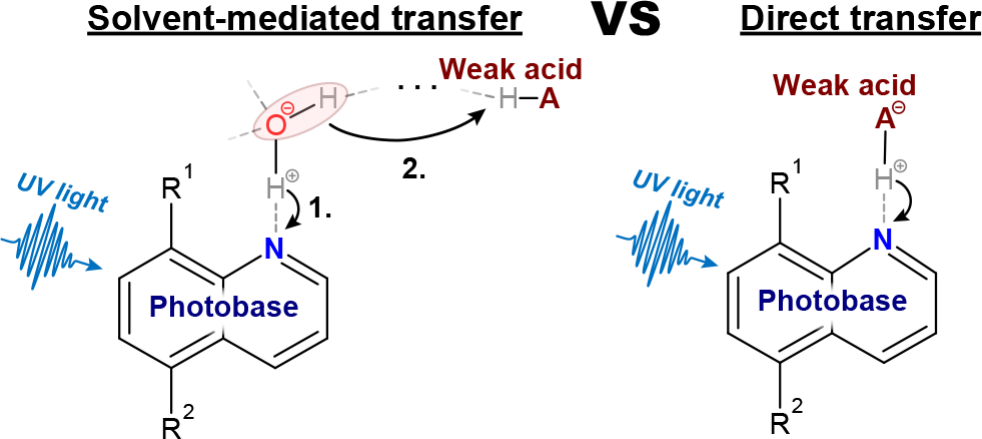

Proton-transfer reactions in water are essential to chemistry and biology. Earlier studies reported on aqueous proton-transfer mechanisms by observing light-triggered reactions of strong (photo)acids and weak bases. Similar studies on strong (photo)base–weak acid reactions would also be of interest because earlier theoretical works found evidence for mechanistic differences between aqueous H^+^ and OH^–^ transfer. In this work, we study the reaction of actinoquinol, a water-soluble strong photobase, with the water solvent and the weak acid succinimide. We find that in aqueous solutions containing succinimide, the proton-transfer reaction proceeds via two parallel and competing reaction channels. In the first channel, actinoquinol extracts a proton from water, after which the newly generated hydroxide ion is scavenged by succinimide. In the second channel, succinimide forms a hydrogen-bonded complex with actinoquinol and the proton is transferred directly. Interestingly, we do not observe proton conduction in water-separated actinoquinol–succinimide complexes, which makes the newly studied strong base–weak acid reaction essentially different from previously studied strong acid–weak base reactions.

## Introduction

Proton transfer in aqueous media plays a crucial role in many fundamental processes in nature. For instance, in living systems, proton-transfer systems regulate essential processes in photosynthesis, including the storage and consumption of energy.^1^ Gradients in proton concentration also drive ion transporters fundamental to cellular life.^2^ Changes in the local proton concentration near solvated proteins can also lead to the (de)protonation of functional groups, potentially causing structural changes and the denaturation of the protein.^3^ In view of the crucial and ubiquitous role of proton-transfer reactions, the better understanding and controlling of these reactions will find many applications. Potential applications involve fuel cells,^4^ optical pH control,^5^ and light-based manipulation of proton conductivity.^6^

In order to gain a molecular-scale understanding of proton-transfer mechanisms, earlier studies examined light-triggered proton-transfer reactions in water^7−11^ and other solvents.^12,13^ Most commonly, these studies made use of weak bases and UV-excited photoacids, which are molecules that enhance their acidity and release a proton upon absorbing light.^14^ By monitoring the proton-transfer dynamics of strong (photo)acids and added weak base reaction partners, aqueous proton-transfer mechanisms became accessible to study.^9−11,15^ The reaction dynamics observed in these studies indicate strong solvent effects, in particular solvent-assisted proton transfer occurring within the spatial range of a few water molecules, through transient “water wires”.^9−11,16^ In view of the many reports of studies on photoacids^9−12,14,15,17^ and photobases,^18−28^ as well as proposed mechanistic differences in aqueous H^+^/OH^–^ transfer,^29−31^ it is surprising that there has been very little work done on similar proton-transfer reactions between strong (photo)bases and weak acids. Because of this, it is currently unknown whether aqueous strong base–weak acid reactions show similar solvent-assisted proton-transfer pathways as strong acid–weak base reactions do.

**Figure 1:**
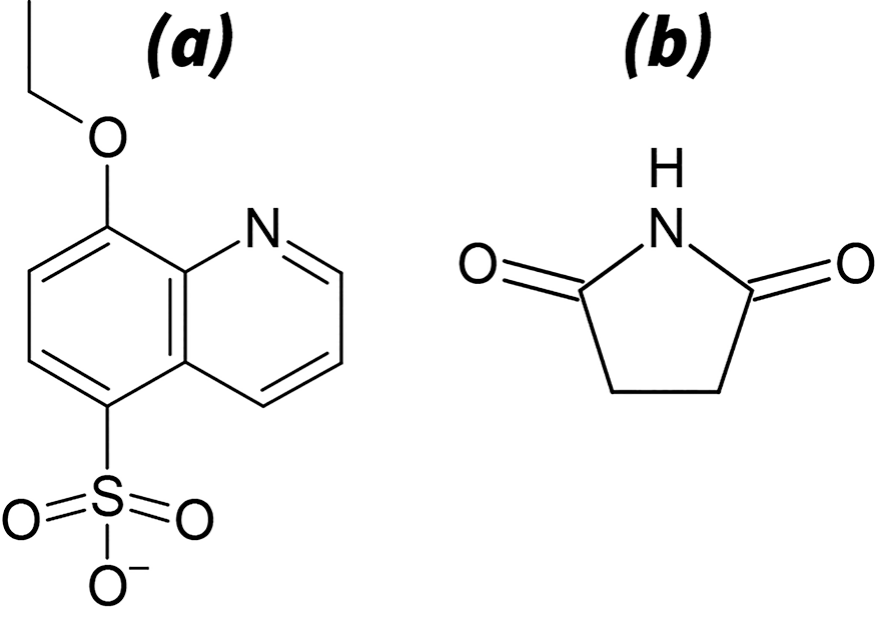
Chemical structures of (a) actinoquinol (AQ^–^) and (b) succinimide (HSI).

To answer the above question of reaction symmetry, we report here on the first study of aqueous proton-transfer mechanisms in strong base–weak acid reactions, enabled by the newly introduced photobase actinoquinol (abbreviated AQ^–^, Figure 1(a)).

AQ^–^ is water-soluble (*c*_*max*_ > 200 mM), shows reversible proton uptake, and is biocompatible.^32,33^ By exciting AQ^–^, we trigger bimolecular proton-uptake reactions, which we monitor using femtosecond UV pump–mid-infrared probe experiments. Here, we added succinimide (HSI, Figure 1(b)) as a weak acid reaction partner. Based on the observations, we develop a generalized model for bimolecular aqueous proton-uptake reactions that includes the role of the solvent. We then compare the results with the characteristics of the proton-transfer reaction between a photoacid and a weak base, i.e., the proton-transfer reaction between HPTS (8-hydroxypyrene-1,3,6-trisulfonate) and acetate.^9^

## Results and Discussion

### Photobasic Properties of Actinoquinol

**Figure 2:**
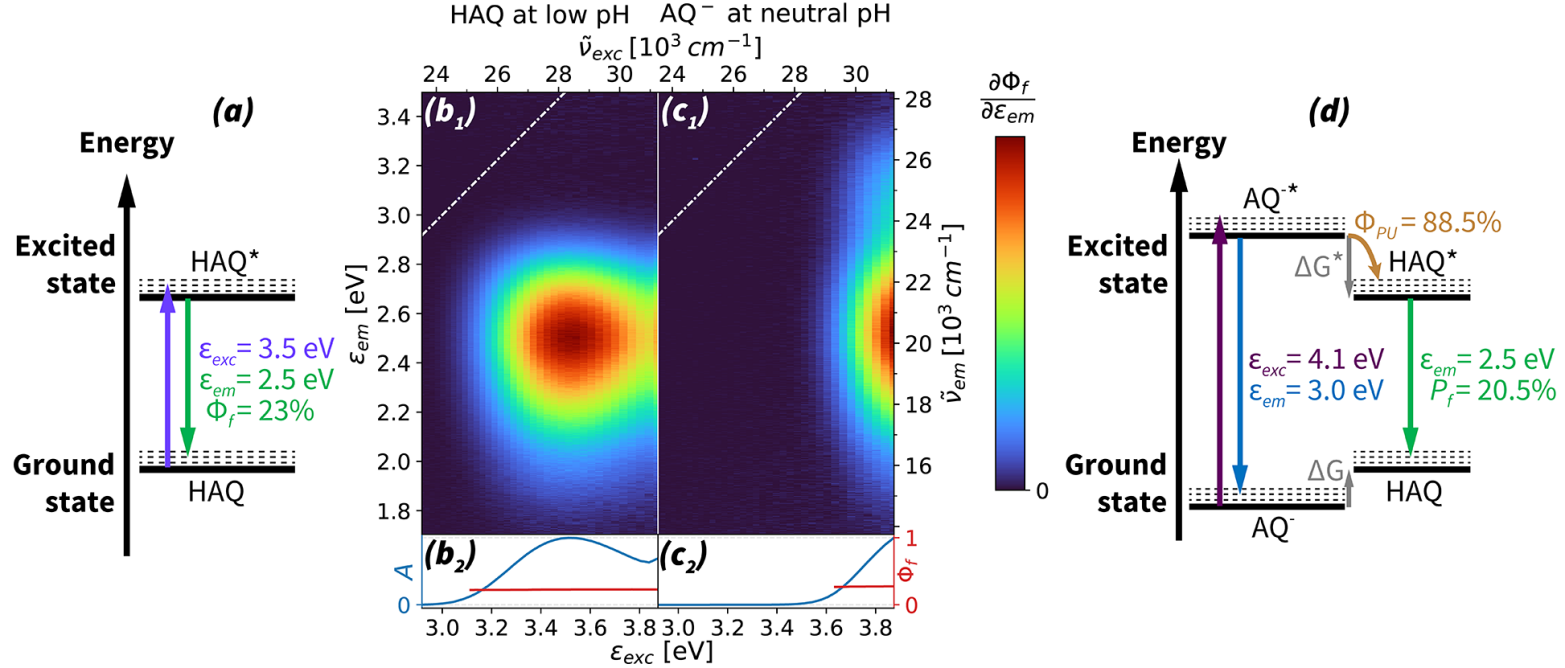
Fluorescent characterization of AQ^–^ and its protonated form, HAQ, in H_2_O. (a) Retrieved Jablonski diagram of HAQ, based on b_1_ and b_2_. (b_1_ and b_2_) Simultaneously recorded absorption (*A*, blue curve), fluorescence quantum yield^34^ (Φ_*f*_, red curve), and emission probability density  spectra (SAFE^35^) of HAQ, recorded in acidified H_2_O. The displayed spectra are normalized and plotted as a function of the respective excitation (ε_*exc*_) and emission photon energies (ε_*em*_) and wavenumbers . The dash-dotted lines in (b_1_) and (c_1_) represent equal excitation and emission energies. (c_1_ and c_2_) SAFE recordings of AQ^–^ recorded in neat H_2_O. Note that two emission bands are observable, one of which coincides with that of HAQ^*^. (d) Jablonski diagram of AQ^–^, including proton uptake from H_2_O. Here, *P_f_* represents the probability of fluorescent relaxation via HAQ^*^. We additionally convert the displayed energy values to wavelengths/wavenumbers; see SI Table 4 and SI Table 5.

In Figure 2, we compare the fluorescence properties of AQ^–^ and its protonated form HAQ (protonated at the nitrogen atom; see SI Figure 1(a)). We show simultaneously recorded UV absorption and fluorescence spectra (SAFE^34,35^) as a function of the excitation energy (ε_*exc*_). By comparing these excitation–emission matrices (EEM) with the absorption spectra, we can rule out any effects from potentially overlapping excited states.^19,36^

Figure 2(b_1_,b_2_) show the SAFE recording of HAQ. We observe that the strongest absorption coincides with the strongest emission. We also find that both the emission profile and quantum yield (Φ_*f*_) are constant within the error of the measurement, at all excitation energies. A very similar behavior is observed for protonated actinoquinol dissolved in D_2_O (DAQ); see SI Figure 4(b_1_,b_2_). Together, these observations indicate that all observed emission around 2.5 eV originates from a single electronic state. We denote this lowest singlet excited state as HAQ^*^ and DAQ^*^. We show the corresponding Jablonski diagrams in Figure 2(a) and in SI Figure 4(a).

Interestingly, the SAFE recordings of AQ^–^ in H_2_O (see Figure 2(c_1_,c_2_)) and in D_2_O (SI Figure 4(c_1_,c_2_)) show that exciting AQ^–^ yields two emission bands, an intense band at 2.5 and a weaker band at 3 eV. Their ratio and yields are excitation independent in both solvents. We thus conclude that these bands result from processes originating in the same excited state accessed by exciting AQ^–^, denoted by AQ^–*^. To separate the two bands, we use emission band profile fitting.^19^ We find that all four emission spectra can be decomposed in just two bands (SI Figure 5). Thus, we assign the 3 eV band to AQ^–*^ and the 2.5 eV band to H/DAQ^*^. The only process that could generate H/DAQ^*^ from AQ^–*^ is the proton-uptake reaction from water, confirming that AQ^–^ is a strong photobase.

Next, we determine the quantum efficiency of the proton-uptake process (Φ_*PU*_) of AQ^–*^. Φ_*PU*_ describes the fraction of AQ^–*^ molecules that extract a proton from water, generating H/DAQ^*^. To calculate this fraction, we divide the probability of fluorescent relaxation (*P*_*f*_) via H/DAQ^*^, obtained from exciting AQ^–^ at neutral pH, and from exciting H/DAQ at low pH. We thus obtain that  in H_2_O and Φ_*DU*_ = 76% in D_2_O, with “*↑*” and “*↓*” representing excitation and fluorescent emission, respectively. These observations together lead to the combined Jablonski diagram of AQ^–^ and H/DAQ as illustrated in Figure 2(d) and in SI Figure 4(d).

**Figure 3:**
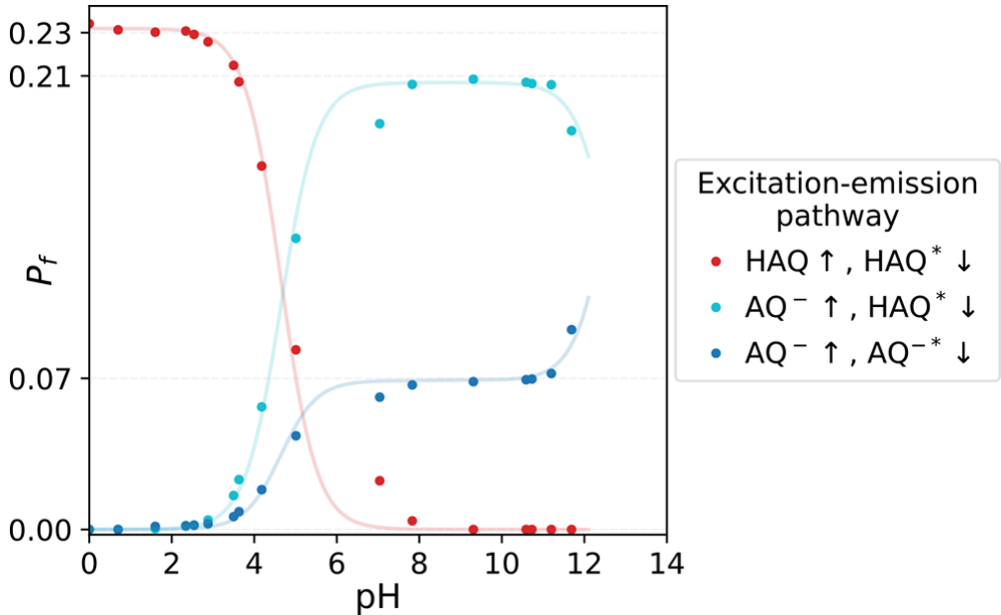
Competition of HAQ^*^ and AQ^–*^ as a function of pH. The probability of fluorescent relaxation (*P*_*f*_) via different excitation–emission pathways is calculated based on band-fitting analysis and quantitative absorption titration. In the legend, “*↑*” and “*↓*” represent excitation and fluorescent emission, respectively. The excitation energy is chosen to match the transient absorption experiments performed at 3.63 eV. Note that the pH measurement is less accurate between pH values 6 and 8. Solid lines are a guide to the eye.

Performing the above analysis at different pHs offers further insight into the properties of the proton-uptake process. Using absorption titration (SI Figure 6), we obtain quantitative ratios of HAQ and AQ^–^ in the ground states (SI Figure 7). Combined with the SAFE analysis, we calculate *P*_*f*_ for all three previously described cases of absorption and fluorescent emission, i.e., excitation of HAQ and emission of HAQ^*^, excitation of AQ^–^ and emission of AQ^–*^, and excitation of AQ^–^ and emission of HAQ^*^. We illustrate the competition of these processes in Figure 3.

Below pH 11, we find that the ratio of the blue and cyan curves, , is constant. Because both of these processes result from light absorption by AQ^–^, their constant ratio implies that Φ_*PU*_ too is pH independent. This indicates that AQ^–*^ does not rely on scavenging solvated protons like a weak photobase would,^37^ but instead directly extracts a proton from a nearby water molecule. At higher pHs, however, we observe shifts in the competition of the excitation–emission pathways. The weakening of the HAQ* relaxation pathway suggests a less efficient protonation reaction. To corroborate this finding, we perform thermodynamic calculations.

Using the Förster-cycle analysis,^19,38,39^ we can correlate the difference of the energy gaps Δ*G* and ΔG^*^ (illustrated in Figure 2(d) and SI Figure 4(d)) with the difference of the p*K*_*b*_ and  values corresponding to AQ^–^ and AQ^–*^. To obtain , we use the p*K*_*b*_ value measured using absorption titration. There, we fitted the fraction of AQ^–^ molecules (SI Figure 7) and obtained that p*K*_*b*_ = 14 – p*K*_*a*_ = 9.85. Applying the Förster-cycle analysis, we obtain that photoexcitation decreases the p*K*_*b*_ of AQ^–^ by 9.1 units; thus  = 0.75. Based on the Marcus-BEBO model,^20,40,41^ we propose that this lower thermodynamic drive causes AQ^–*^ to protonate slower than other quinolines, such as 5-methoxyquinoline ( = −1.1) or 5-aminoquinoline ( = −1.9).^22,24^

The analysis of the fluorescence quantum yields also offers further insight into the isotope effect of the proton uptake. In the case of AQ^–*^ ions in neat water, the excited-state dynamics are governed by competing first-order processes. Assuming a one-way proton-uptake reaction, we can relate the proton extraction rate (*k*_*PU*_) of AQ^–*^ to its fluorescent relaxation rate  by comparing their yield ratios. In H_2_O, we obtain , and in *D*_2_O, .

As the fluorescent relaxation rate of blue emitters is typically very similar in H_2_O and in D_2_O,^42^ we can calculate the kinetic isotope effect: *k*_*PU*_:*k*_*DU*_ = 3.2:1. This ratio is very close to earlier reports for 6-methoxyquinoline, which is another quinoline with a similar base strength ( = 2.2).^18^

The quantum yield measurements also allow for an estimation of the efficiency of retaining protons beyond the singlet lifetime of HAQ^*^. According to our measurements, the emission yields of HAQ^*^ and DAQ^*^ are quite different; namely,  and . Based on earlier works,^43−46^ we propose that this striking difference originates from an isotopic difference in the balance of fluorescent relaxation and other nonradiative relaxation methods, such as intersystem crossing (ISC) to triplet states. ISC was found to be fairly common for quinolines,^24^ and its rate was also shown to be sensitive to isotope effects due to vibronic couplings.^47^ If ISC is indeed the cause of the emission yield difference, then the majority of the HAQ^*^ molecules would relax via long-lived triplet states. Having both efficient and long-lived proton extraction capabilities while being water-soluble makes actinoquinol a highly suitable molecule for optical pH control.

### Dynamics of Proton-Uptake Reactions

**Figure 4:**
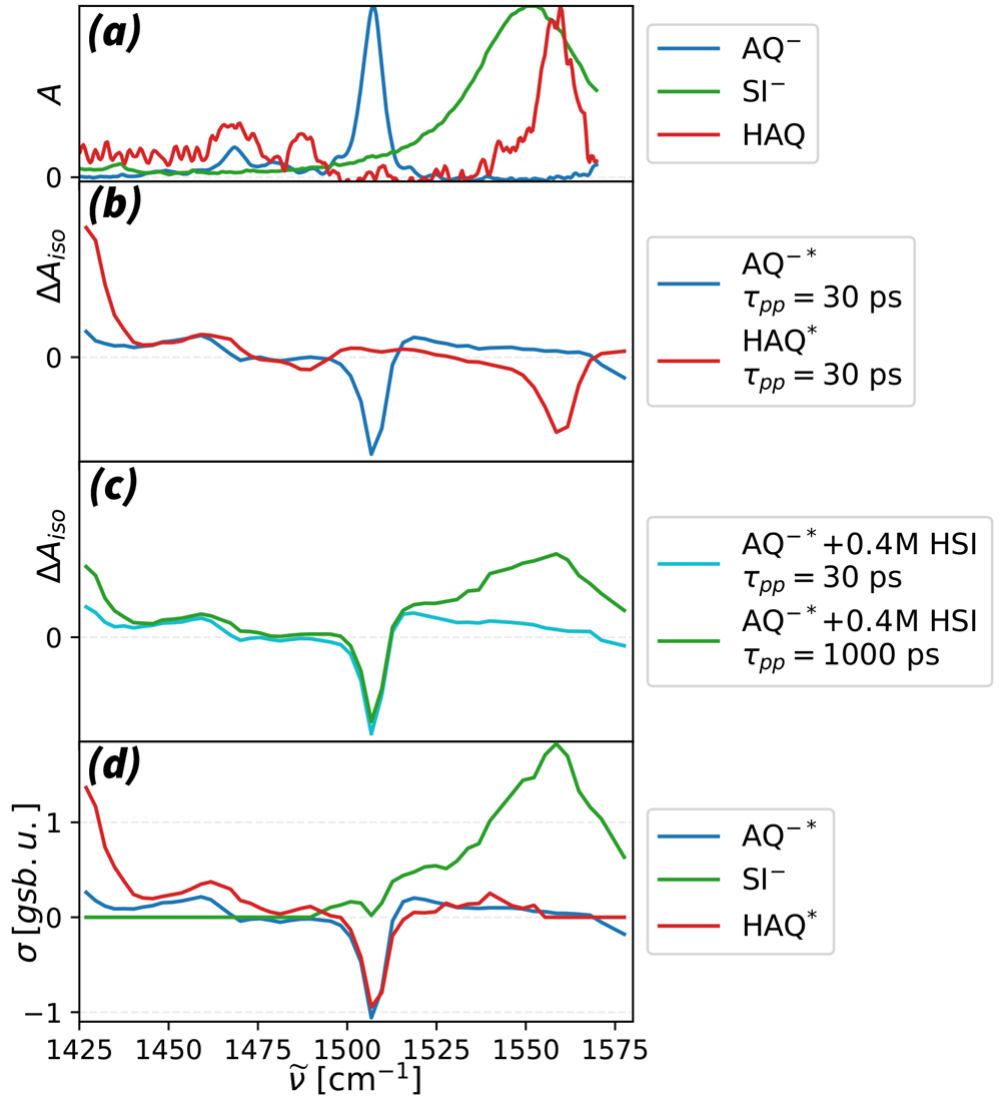
(a) Normalized steady-state infrared absorption spectra (*A*) of AQ^–^, SI^–^, and HAQ with solvent features subtracted, plotted as a function of spatial frequency (ν̃). HSI does not have significant features in this region. (b) Normalized isotropic transient absorption spectra (Δ*A*_*iso*_) of AQ^–^^*^ and HAQ^*^ at τ_*pp*_ = 30 ps. (c) Δ*A*_*iso*_ for AQ^–^^*^ in a solution of 0.4 M HSI in H_2_O, at τ_*pp*_ = 30 ps and at τ_*pp*_ = 1000 ps, showing emerging HAQ^*^ and SI^–^ features. (d) Spectral components (σ) resulting from soft kinetic modeling of the transient absorption data. The amplitudes are normalized to the ground-state bleach of AQ^–^^*^ at 1507 cm^–1^ shortly after excitation, which thus equals 1 ground-state bleach unit (gsb.u.).

Next, we study the reaction of photoactivated AQ^–^ with a weak acid. We chose succinimide as the reaction partner because it possesses several favorable properties. First, succinimide is a neutral molecule with a small static dipole;^48^ thus we avoid strong electrostatic effects. It is also well-soluble in water (*c*_*max*_ > 2 M). Third, succinimide has strong and unique infrared features while being transparent at the frequencies of almost all AQ^–^ and HAQ vibrations (see Figure 4(a) and main feature assignment in the SI). Finally, succinimide has a p*K*_*a*_ of 9.56,^49^ which is higher than the ambient pH of water, but is still a much stronger acid than HAQ^*^, with . These properties together mean that we can observe and monitor the reaction of AQ^–*^ and succinimide in neat water.

To study the bimolecular reaction dynamics, we performed transient absorption (TA) measurements at various succinimide concentrations, both in H_2_O and in D_2_O. To initiate the reaction, we excited AQ^–^ in solutions with and without succinimide, using a 300 fs laser pulse centered at 3.63 eV (342 nm, 29200 cm^–1^). At this wavelength AQ^–^ absorbs while H/DSI does not (SI Figure 6). We used a low pump intensity and a rotating sample cell, to prevent photodegradation and to ensure continuous sample refreshment. A complete description of our experiment can be found in the SI.

In Figure 4(a), we observe that deprotonated succinimide (SI^–^, see SI Figure 1(b)) has a strong and broad absorption peak at 1557 cm^–1^, corresponding to its asymmetric C=O vibration.^50^ We use this feature to monitor the proton release by succinimide in the TA experiments.

The signals of the proton acceptor arise at different frequencies. Based on Figure 4(b), we first assign features of AQ^–*^ (blue) at τ_*pp*_ = 30 ps, when solvent relaxation is complete^51^ (see SI Figure 12). We observe negative transient absorption signals at the frequencies where ground-state AQ^–^ absorbs. These signals are thus attributed to ground-state bleaches. The positive transient absorption signals represent the excited-state absorption of AQ^–*^. A similar analysis for HAQ^*^ (red) suggests that we can expect signatures of protonation at 1425 cm^–1^ and near 1465 cm^–1^. When dissolved in neat H_2_O, we find that the 1425 cm^–1^ feature is rising on the nanosecond time scale. Conducting a similar analysis on the D_2_O measurement series (shown in SI Figure 13), we find that the AQ^–*^ signals are identical after solvent relaxation and that the deuteration of AQ^–*^ to DAQ^*^ is leading not only to a 1425 cm^–1^ feature but also to a feature centered at 1490 cm^–1^.

**Figure 5:**
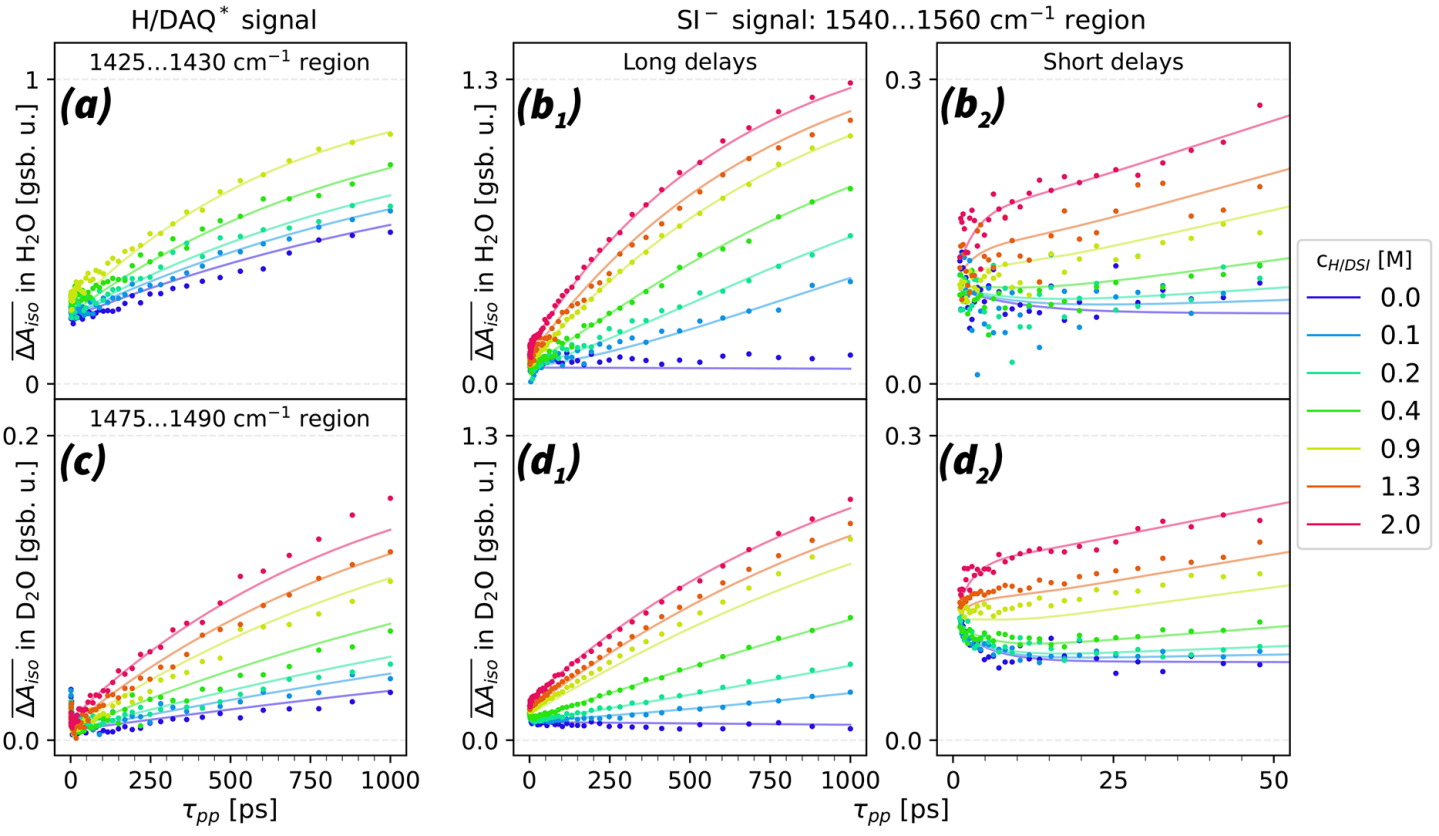
Transient absorption signal as a function of pump–probe delay (τ_*pp*_) for different succinimide concentrations (*c*^*H*/*DSI*^). (a) Spectrally averaged transient absorption dynamics  of HAQ^*^ in the 1425–1430 cm^–1^ region for solutions in H_2_O. (b_1_, b_2_) Transient absorption in the 1540–1560 cm^–1^ spectral region in H_2_O. This spectral region almost exclusively represents the response of SI^–^. The minor signal decrease at low *c*^*HSI*^ and τ_*pp*_ < 20 ps can be explained from solvent relaxation effects following the generation of AQ^–^^*^. (c) Transient absorption of DAQ^*^ in the 1475–1490 cm^–1^ region, for solutions in D_2_O. (d_1_, d_2_) Transient absorption in the 1540–1560 cm^–1^ spectral region in D_2_O. The presented experimental plots are normalized to an identical initial AQ^–^^*^ population. The solid lines represent fits to the data using the kinetic reaction model described in the text.

In order to understand the overall reaction kinetics, we analyze the TA dynamics in Figure 5. We made the TA signals directly comparable by matching them at early delays. We detail our full analysis approach in the SI.

In Figure 5(b_1_,b_2_), we plot signals that correspond to the main features of succinimide in H_2_O. We do the same in Figure 5(d_1_,d_2_) for the corresponding signals in D_2_O. In Figure 5(a) and (c) we additionally present the transient signals corresponding to the creation of HAQ^*^ and DAQ^*^. Note that the primary 1425 cm^–1^ features of H/DAQ^*^ can only be observed well for solutions with *c*^*H*/*DSI*^ < 1 M, because H/DSI weakly absorbs at ≈1428 cm^–1^ (SI Figure 10(c)). Therefore, we can monitor the creation of HAQ^*^ features in this concentration range. The production of DAQ^*^, however, can be monitored at all DSI concentrations, using its transient absorption feature at 1490 cm^–1^.

In Figure 5(a) and (c), we compare the generation dynamics of HAQ^*^ and DAQ^*^. In the absence of succinimide, we observe a steady creation of H/DAQ^*^ in H/D_2_O. This generation is the result of the AQ^–*^ + H/D_2_O → H/DAQ^*^ + OH/D^–^ reaction, in agreement with the fluorescence measurements. The almost linear rise of the H/DAQ^*^ signals indicate that the time constants of these reactions are longer than the 1 ns observation window. In addition to the nanosecond dynamics of the HAQ^*^ and DAQ^*^ signals, we also observe a small signal decay on the 10 ps scale; see SI Figure 12. We attribute this signal to solvent relaxation effects following the generation of AQ^–*^.^51^

In Figure 5(a) and (c), we observe that upon adding succinimide, the H/DAQ^*^ generation accelerates, and that this acceleration is most pronounced at *c*^*HSI*^ > 0.4 M. This acceleration indicates that AQ^–^^*^ reacts directly with succinimide, in addition to its reaction to H/D_2_O. The direct AQ^–^^*^–DSI reaction in D_2_O appears to outcompete the AQ^–^^*^–D_2_O reaction at *c*^*DSI*^ > 0.2 M.

In Figure 5(b_1_), we also compare the generation dynamics of SI^–^ in H_2_O for different concentrations of HSI. At *c*^*DSI*^ ≤ 0.2 M, the SI^–^ signal does not rise immediately like the HAQ^*^ signal, but in a delayed manner. Adding more succinimide enhances the SI^–^ signal and makes its dynamics more similar to the HAQ^*^ signal. We additionally observe that the reaction saturates with increasing SI^–^ concentration. This saturation effect can be illustrated by comparing the SI^–^ signals at 1 ns, measured at concentrations of 0.4 and 2 M: the 5-fold concentration increase only changes the observable signal by approximately 50%. A similar analysis in D_2_O reveals comparable reaction dynamics, with the difference that the DAQ^*^/SI^–^ signals already show more similar dynamics at *c*^*DSI*^ ≈ 0.2 M (see Figure 5(d_1_)). This similarity shows that the direct AQ^–*^–DSI reaction dominates at *c*^*DSI*^ > 0.2 M.

Finally, we observe an additional, quickly rising signal of succinimide at τ_*pp*_ < 25 ps (see Figure 5(b_2_) and (d_2_)). This signal shows fast dynamics and a near-quadratic amplitude dependence with increasing succinimide concentration.

### Modeling and Discussion

#### Transient Species Identification Using “Soft” Kinetic Modeling

To analyze our transient absorption signals, we first identify spectral components of the transient chemical species using a purpose-developed approach, which we denote as “soft” kinetic modeling. We then use the thus determined spectral components to identify the physical interactions and elementary steps of the reaction mechanism leading to the observed dynamics.

In the “soft kinetic modeling” approach, we distinguish spectral components based on simple, physical assumptions. Such assumptions include constraints on spectral shapes or on the distribution of reactant populations at τ_*pp*_ = 0, or enforcing a constant-rate reaction with the solvent (water), or the conservation of mass between different chemical species (e.g., to describe mass transfer in reactions from AQ^–*^ to H/DAQ^*^). To achieve the best fit to the observed TA signals, we account for the reaction dynamics using multiexponential functions. The resulting fits are numerically accurate, even though such reaction rates are not derived from deeper physical considerations. We present a full description in the SI.

Using this method, we separated three main and two auxiliary spectral components in H/D_2_O. These main components are assigned to AQ^–*^, H/DAQ^*^, and SI^–^. The auxiliary components correspond to presolvated AQ^–*^ and to the red-shift of the 1507 cm^–1^ feature of AQ^–^ in the AQ^–^···H/DSI complexes. Using just these components, we could accurately reconstruct all observed TA signals; see an example in SI Figure 17. Next, we show the main spectral signatures (Figure 4(d) and in SI Figure 13(d)). As expected, the spectrum of the AQ^–*^ component is identical to the TA spectrum of AQ^–*^ in H_2_O at short delays. The HAQ^*^ component also closely resembles the spectrum obtained by exciting HAQ. Last, we find that the SI^–^ spectral component is also very similar to its steady-state signature. Having repeated this analysis for D_2_O-based measurements, we find that the AQ^–*^ and SI^–^ components in D_2_O are both very similar to those in H_2_O; see SI Figure 13(d).

#### Kinetic Reaction Modeling

Using the spectral components that constitute the TA signals at all delays, we then develop a kinetic model that describes the different reaction pathways. One of the reaction pathways accounts for the AQ^–*^ + H/D_2_O → H/DAQ^*^ + OH/D^–^ reaction, which is governed by *k*_*P*/*DU*_. The generated OH/D^–^ ions can be subsequently scavenged by succinimide following the OH/D^–^ + H/DSI → H/D_2_O + SI^–^ reaction. We assume that during this acid–base neutralization reaction the succinimide molecules are evenly distributed in the bulk. Therefore, we propose that this reaction is governed by the bimolecular rate constant *k*_*neut*_.

Next, we consider the direct reaction between AQ^–*^ and succinimide. Based on the increasing correlation of H/DAQ^*^ and SI^–^ signals, we established that the contribution of the direct reaction is increasing with succinimide concentration and that it dominates the reaction in D_2_O at *c*^*DSI*^ > 0.2 M. To explain the strong saturation of the H/DAQ^*^ and SI^–^ signals, we propose that the direct reaction between AQ^–*^ and H/DSI occurs in hydrogen-bonded AQ^–*^···H/DSI complexes. The formation of these complexes will saturate with increasing succinimide concentration and is also observable in the ground state. Earlier works show a red-shift in the UV absorption spectrum of other quinolines^52^ and photoacids^16^ upon changing their hydrogen-bonding partner at the reactive site. AQ^–^ shows a similar red-shift upon adding succinimide, which saturates with increasing succinimide concentration (SI Figure 6). Because succinimide does not absorb in this region (SI Figure 6), we conclude that this red-shift indicates the ground-state formation of AQ^–^···H/DSI hydrogen-bonded complexes, similar to those between HPTS and acetate.^16^ By performing quantitative analysis of this red-shift (SI Figure 9), we obtained the association constant . To describe the reaction rate of these AQ^–^···H/DSI complexes, we use the rate constant *k*_*direct*_. Finally, we also account for the enhanced absorption cross-section (σ) of the AQ^–^···H/DSI complexes at the pump wavelength of 342 nm by including their preferential excitation in our calculations of initial AQ^–*^ populations.

We find that using only the effects described above (SI Figure 19), we cannot accurately fit our data (SI Figure 20), especially at longer delays and higher concentrations. This is because in this model the associated pairs quickly deplete, and therefore the long-delay dynamics will only be determined by the reaction pathway with OH/D^–^. This pathway is limited by the production rate of OH/D^–^, determined by the reaction of AQ^–^^*^ with H/D_2_O. To obtain a more accurate description, we consider that there will be an ongoing production and dissociation of hydrogen-bonded complexes of AQ^–^^*^ and H/DSI molecules, governed by the rate constants *k*_*assoc*_ and *k*_*dissoc*_. To estimate these rate constants, we assume that the reorientation of AQ^–^^*^ in water is accompanied by the reorganization of the surrounding hydrogen-bonding network. In the case of hydrogen-bonded AQ^–^^*^···HSI pairs, this will also likely lead to the breaking of the hydrogen-bond between the two. We therefore took the rate constant *k*_*dissoc*_ to be equal to the anisotropy decay rate, *k*_*ani*_. This rate should be very similar for AQ^–^^*^ and H/DAQ^*^, which we can accurately measure using polarization-resolved measurements (see SI Figure 15). We thus obtain the association rate constant: .

Last, we also account for the quickly reacting subpopulation of the hydrogen-bonded AQ^–*^···H/DSI complexes. Earlier, we observed that their TA signal amplitude at τ_*pp*_ < 25 ps shows a negligible isotope dependence and increases approximately  at lower concentrations. A possible explanation for this fast component is that it occurs in complexes formed by one AQ^–*^ and two HSI molecules, i.e., AQ^–*^(H/DSI)_2_ complexes, referred to as trios. The quadratic concentration dependence of the amplitude of the fast component emerges naturally if we consider the secondary association constant: . At low *c*^*H/DSI*^, *c^AQ^−^···HSI^∝ c^H/DSI^*; thus we obtain that . To describe the reaction rate of these quick reaction complexes, we introduce *k*_*trio*_. We find that we get the best fits if both  and *k*_*trio*_ are very similar for H_2_O and D_2_O. Because the contribution of the trios is altogether rather small and we also lack information about their association dynamics, we did not include these complexes in the dynamic association–dissociation process.

**Figure 6:**
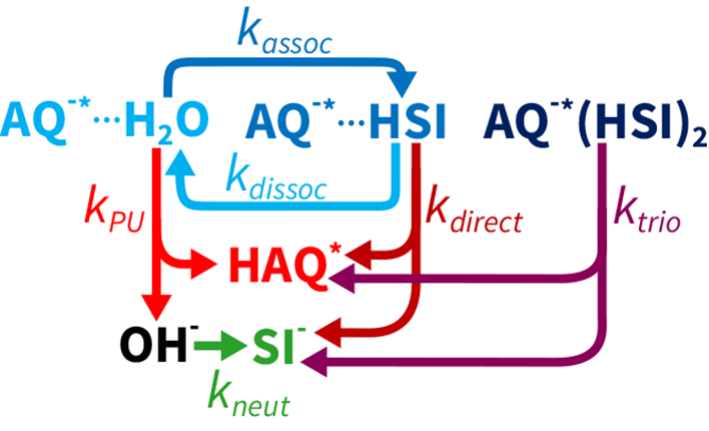
Graphical representation of the different reaction pathways for the reaction of AQ^–*^ with H/D_2_O and H/DSI. The different chemical species are illustrated with labels. The connecting arrows denote the reaction pathways, while matching labels describe their rate. For the clarity of illustration, we omitted the excited-state decay pathways. The full rate-matrix description including these pathways is presented in eq 3 of the SI.

The kinetic model is illustrated in Figure 6, and the explicit mathematical formulation of this model is presented in the SI. With this model, we obtain an excellent fit of the transient absorption signal at all concentrations and delay times, as shown in Figure 5. The resulting fit parameters are presented in Table 1.

**Table 1:** Model Parameters Providing the Best Fit of the Observed TA Dynamics^a^

parameter	in H_2_O	in D_2_O	unit
*k*_*dissoc*_^–1^	60.4	61.5	ps
*K*_*assoc*_^*pair*^	0.47	0.51	M^–1^
*k*_*assoc*_^–1^	131.5	130.7	ps M
σ^*assoc*^:σ^*free*^	1.58:1	1.54:1	-
*k*_*PU*_^–1^	2.46	7.9	ns
*k*_*neut*_^–1^	50	130	ps M
*k*_*direct*_^–1^	360	570	*ps*
*k*_*trio*_^–1^	3	3	ps
*K_assoc_^trio^*	0.045	0.05	M^–2^

a Displayed absorption cross-sections are measured at 3.63 eV, the excitation photon energy in the UV pump–mid-infrared probe experiments. We provide error estimates for *k*_*PU*_, *k*_*direct*_, and *k*_*neut*_ in the SI.

Based on the retrieved parameters, we find that some are isotope-dependent. One is the rate of the proton-uptake reaction, which has a KIE of 3.2:1, which we obtained using SAFE measurements. Additionally, the hydroxide–succinimide neutralization reaction shows a similarly large KIE of 2.6:1. The direct reaction between AQ^–^^*^ and succinimide, however, has a much smaller KIE of 1.6:1, which is half the KIE of the reaction with water.

**Figure 7:**
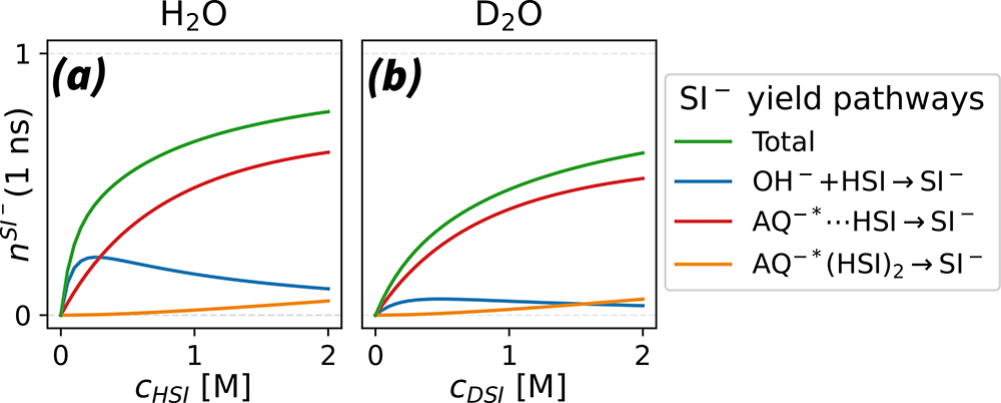
SI^–^ populations created via different reaction pathways during the first nanosecond of the bimolecular reaction ((1 ns)), plotted as a function of succinimide concentrations (*c*^*H*/*DSI*^). These SI^–^ populations are calculated using the respective fitting parameters for the reaction in H_2_O (a) and in D_2_O (b) and are normalized to the initial AQ^–*^ population ((0 ns)). We show the total SI^–^ yield (green) and contributions from the OH^–^ neutralization reaction pathway (blue) and from the direct AQ^–^ reaction pathway in different associated subspecies, i.e., in pairs (red) and in trios (orange).

We highlight the reaction trends in Figure 7. Here, we calculated the proportion in which different reaction pathways contribute to the rise of the SI^–^ signal.

In Figure 7(a) we find that in H_2_O the OH^–^ neutralization reaction pathway is dominant only at low succinimide concentrations, with a maximum at approximately *c*^*HSI*^ = 0.25 M. Here, the rate-determining step is the scavenging of the generated OH^–^ by HSI molecules. This is corroborated by Figure 5(a) and (b_1_), which show that the rise of the SI^–^ signal lags behind the rise of the HAQ^*^ signal at *c*^*HSI*^ = 0.1 M, due to the delay induced by the second scavenging step. According to Figure 7(a), this channel starts declining around *c*^*HSI*^ = 0.3 M, which suggests that the direct transfer pathway becomes dominant. This is in agreement with the UV absorption measurements, which indicate that at a concentration of *c*^*H*/*DSI*^ = 2 M the fraction of AQ^–^ molecules forming hydrogen-bonded complexes with H/DSI (*r*^*assoc*^) reaches a level of ≈50%.

The competition between the direct and the scavenging channel is somewhat different in D_2_O; see Figure 7(b). Here, we find that the OD^–^ scavenging reaction pathway has a rather small contribution, mainly due to the much slower OD^–^ generation. As such, the direct pathway dominates already at low DSI concentrations.

Finally, we compare our results for the reaction between the strong photobase AQ^–^ and the weak acid succinimide with the well-studied reaction between the strong photoacid HPTS and the weak base acetate (Ac^–^). Their thermodynamic drives^53,54^ of  and  mirror the drives of the AQ^–^–HSI system very well, with  and .^49^ Despite these similarities, we find a striking difference between the rate constants with the solvent, with  = 90 ps (210 ps) for HPTS^*^^55^ (DPTS^*^^56^) and  = 2.5 ns (7.9 ns) for AQ^–*^ in H_2_O (D_2_O). Additionally, the rate of pair reactions is also much higher for HPTS^*^ (DPTS^*^): in hydrogen-bonded Ac^–^···DPTS^*^ pairs,  100 fs,^9,10^ while in AQ^–*^···HSI (DSI) pairs,  = 360 ps (570 ps). Moreover, these are not the only differences: the mechanism of the proton-transfer reaction is also rather distinct, as we do not find evidence for direct proton transfer in solvent-separated AQ^–*^/HSI pairs, unlike in solvent-separated DPTS^*^/Ac^–^ pairs.

This difference in the mechanism of direct proton transfer between the DPTS^*^/Ac^–^ and the AQ^–*^/HSI systems may be related to differences in aqueous H^+^/OH^–^ transport mechanisms. Earlier MD studies^29−31^ suggest that hydroxide’s first hydration shell has a different configuration, which is rather stable, as it might involve supercoordination with four nearby water molecules. This is different from the hydration shell of protons, which rapidly interchange between Eigen-like and Zundel-like configurations.^57−59^ Consequently, hydroxides are likely subject to stepwise propagation^29,30^ or shorter hops limited to one or two molecules,^31^ as opposed to the multimolecular hops of hydrated protons. Other works,^60^ however, suggest that the H^+^/OH^–^ transport might not be so different.

Additionally, the difference in direct proton-transfer mechanisms in the DPTS^*^/Ac^–^ system and the AQ^–*^/HSI system may also be due to the different time scales of their proton-transfer reactions. For the DPTS^*^/Ac^–^ system, direct proton transfer is relatively fast, taking place on a time scale ranging from sub-picoseconds to tens of picoseconds, depending on the number of intervening water molecules.^9,11^ As a result, the reactions between DPTS^*^ and acetate largely take place in a distribution of systems where the distance between the reactants is more or less static. The AQ^–*^/HSI system, however, is in a quite different limit. Even for directly hydrogen-bonded AQ^–*^/HSI pairs, direct proton transfer is relatively slow, showing a time constant of 360 ps. In view of the distance-dependent slowing of the proton transfer in the DPTS^*^/Ac^–^ system, the direct reactions in AQ^–*^/HSI complexes containing intervening water molecules will likely happen on a time scale of several nanoseconds. Compared to this time scale, the diffusion of HSI is much faster. Hence, for an AQ^–*^/HSI system with intervening water molecules, proton transfer over water wires may in principle be possible but is not observable, as it is completely outcompeted by diffusion, hydrogen-bonded complex formation, and subsequent proton transfer within the established complex.

## Conclusions

Using simultaneously recorded absorption and fluorescence emission measurements, we found that actinoquinol (AQ^–^) is an efficient water-soluble photobase with a high proton-extraction yield. We found that this excited-state reaction with water has a strong isotope effect, and is approximately 3.2 times faster in H_2_O than in D_2_O. By analyzing the fluorescent relaxation probabilities of protonated actinoquinol (HAQ^*^/DAQ^*^), we found evidence that in H_2_O, HAQ^*^ retains its proton beyond its singlet excited-state lifetime; which makes actinoquinol a desirable candidate for optical pH control.

We then used femtosecond UV pump - IR probe spectroscopy to study the rate and mechanism of proton uptake by photo-activated actinoquinol (AQ^−*^) from water. We found that this reaction proceeds with a rate of  = 2.5 ± 1 ns in H_2_O, and and with  = 7.9 ± 3.2 ns in D_2_O. We then also studied the aqueous reaction mechanisms of AQ^−*^ with weak acid succinimide (HSI/DSI). We found that this reaction proceeds via two parallel reaction channels that compete with each other. In the first channel, AQ^–*^ takes up a proton from water, and the newly generated hydroxide ion is scavenged by succinimide. In the second channel, the proton is directly transferred from succinimide to AQ^–*^ in hydrogen-bonded AQ^–*^···H/DSI complexes. This latter mechanism dominates at higher succinimide concentrations and is approximately 7 (14) times faster than the reaction with H_2_O (D_2_O). We additionally found that this second, direct channel contains a very fast component with a contribution that rises approximately quadratically with the succinimide concentration. We inferred that this reaction is likely happening within associated trios of AQ^–*^ and two succinimide molecules, i.e., AQ^–*^(H/DSI)_2_ complexes.

We summarized these findings in a reaction model and found that this model provides an accurate description of the observed reaction dynamics. This means that we found no indication of proton transfer occurring over a distance, e.g., via water wires connecting actinoquinol with succinimide. This, according to earlier studies, makes the proton-transfer mechanism essentially different from that between strong photoacids (e.g., HPTS) and weak bases (e.g., the acetate ion). We thus found evidence that the mechanism of aqueous proton transfer in strong base–weak acid reactions does not mirror that in strong acid–weak base reactions with similar thermodynamic drives. This difference can probably be explained from the relatively low reaction rates of the hydrogen-bonded AQ^–*^/HSI system. We find that  = 360 ps and  = 570 ps. Hence, for the AQ^–*^/HSI system, proton transfer over a distance will happen very slow, requiring several nanoseconds. Compared to this time scale, the diffusion of HSI is much faster. As a result, for the AQ^–*^/HSI system, proton transfer over water wires is likely not observable because it is completely outcompeted by diffusion, hydrogen-bonded complex formation, and proton transfer within the complex. To fully conclude on the role of other effects, e.g., the differences in molecular-level transport mechanisms of hydroxide ions and protons, future experiments are required using stronger photobases showing faster reactions.

## Supporting Information Available

Description of experimental details, data processing approaches, and additional measurements (PDF)
