# Supplementary material for: Observing Aqueous Proton-Uptake Reactions Triggered by Light
Source: J Am Chem Soc. 2023 Mar 20;145(12):6682–90. doi: 10.1021/jacs.2c11441 (PMC10064335; doi:10.1021/jacs.2c11441)
Supplement: Supplementary file 1 — ja2c11441_si_001.pdf [file ja2c11441_si_001.pdf]

Observing aqueous proton-uptake reactions triggered by light

Balázs Antalicz,* Jan Versluis, and Huib J. Bakker*

AMOLF, Ultrafast Spectroscopy, Science Park 104 Amsterdam, Netherlands

E-mail: antalicz@amolf.nl; bakker@amolf.nl

Samples and preparation

Chemicals

We obtained purified H_2O from a Simplicity Millipore system, with a resistivity of 18.2 MΩ·cm. We purchased additional chemicals from Sigma Aldrich/Merck, TCI Chemicals Europe and Honeywell/Fluka. These chemicals were used as received, listed in Table 1. In general, all chemicals were stored in closed containers, in the dark, under nitrogen atmoshpere.

Table 1: List of chemical compounds used in our measurements.

Name	Origin	Form
D_2O	Merck	99.9 atom% D, liquid
NaOH	Honeywell/Fluka	1 mol/l solution
NaOD	Merck	40 wt% solution
HCl	Merck	Fuming, 37 wt% solution
DCl	Merck	35 wt% in D_2O, >99 atom% D
$HClO_4$	Fluka	1 mol/l solution
$DClO_4$	Merck	Fuming, 68 wt% solution, 99 atom% D
Actinoquinol sodium salt*	TCI	n·hydrate powder
Succinimide	TCI	>98% Powder
Rhodamine 6G	TCI	Powder
Fluorescein	Merck	Powder
2,7 Dichloro-fluorescein	Merck	Powder
Coumarine 153	Merck	99% powder
Quinine hemisulfate	Merck	Monohydrate powder
Pyranine	Merck	> 96% powder

*: We received actinoquinol with an unknown amount of crystal water. Therefore, in our calculations we used a molar mass of $M^{NaAQ+n \cdot H_2O} = 320\ \frac{g}{mol}$. All reported concentrations and attenuation coefficients of AQ^- and H/DAQ are derived from this value.

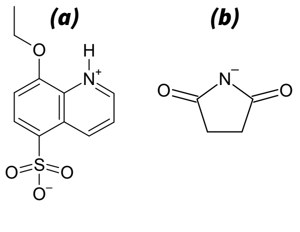

Figure 1: Molecular structures of (a) protonated actinoquinol (HAQ) and (b) deprotonated succinimide (SI^-).

Sample preparation methods

We prepared samples by dissolving the solid compounds to create high-concentration solutions. We then mixed these solutions in predetermined amounts to create solutions with the desired concentrations. Because we used a variety of settings, we note the solutions used in the corresponding section of the SI. Also, since most of the employed molecules absorb UV light, we prepared all our samples under a blue- and UV-free light source, so that we could prevent any possible photodegradation. We note that for each reported solutions of AQ^- or AQ^- with H/DSI, we additionally added a small, calculated amount of NaOH/OD to ensure that the solution pH is kept within the pH 6..8 range. We then verified this using UV-VIS spectroscopy (see the section UV-VIS measurements), where calculate the pH of the solution based on the observed ratio of HAQ and AQ^-.

In this work, we often report volumetric concentrations of succinimide. Establishing the accurate concentration of succinimide is important for modelling purposes. We thus calculated sample concentrations based on solution densities, using the method below.

First, we calibrated our pipettes using H2O and D_2O. We do this by using a Sartorius CP64 analytical scale and a 200 ul Gilson Pipetman pipette. Here, we determine the weight of both H_2O and D_2O for a nominal sample volume 3 × 0.2 ml. We then calculate the densities of both solvents, and compare it to those reported in literature. We thus obtain a volumetric scaling factor (S_V) for the pipette, connecting the nominal and true volumes: $S_V = \frac{1}{2} \cdot \left(\frac{\rho_{lit.}^{H_2O}}{\rho_{exp.}^{H_2O}} + \frac{\rho_{lit.}^{D_2O}}{\rho_{exp.}^{D_2O}} \right)$. Using this correction factor, we calculated the densities of succinimide samples of different concentrations, based on a nominal volume of 0.2 ml. We find a linear increase of the density of succinimide solutions (ρ) with increasing succinimide molality (b) at molalities less than 3 $mol \cdot kg^{-1}$ in H_2O and 2.7 $mol \cdot kg^{-1}$ in D_2O: $\rho(b) = \rho(b = 0) + \alpha \cdot b$. We report our best fit values in Table 2. By recording the mass of components in every solution we created, we could then use this density correlation to calculate the volumetric concentration of succinimide.

Table 2: Best fitting values for densities of H/DSI solutions.

Fit constant	HSI	DSI	Unit
α	0.01668	0.01865	$g \cdot ml^{-1} \cdot mol^{-1} \cdot kg$
ρ_0	0.999	1.100	$g \cdot ml^{-1}$

Additionally, we measured densities of NaAQ solutions as well. We found that with a molality of ≈ 0.1 m, the solution density is identical compared to that of neat H/D_2O within the error of the measurements. As such, we report concentrations of AQ^- by calculating its molar amount per solvent volume. We also neglect the effect of added NaAQ on the density in mixed NaAQ - H/DSI solutions, where instead we use the density of pure H/DSI solutions.

Fluorescence measurements

Experimental methods

We performed simultaneously recorded absorption and fluorescence emission (SAFE[1–3]) measurements using a HORIBA Duetta-Bio. Absorption spectra are collected in a transmission geometry, with a reference photodetector sampling light before the sample position. In addition to referencing absorption measurements, this detector is also used to reference fluorescence measurements, and thus is factory-calibrated to output relative intensities measurable at the sample position.

During fluorescence measurements, emitted light is collected at a right-angle configuration. The dispersed emission spectra are measured using a CCD detector. In order to provide a uniform relative spectral response, HORIBA obtained calibration factors using a combination of NIST intensity correction standards and calibrated light sources.

Typical settings of the physical hardware included 3 nm bandwidth in both excitation and emission paths, in the 300-800 nm spectral region with 3 nm steps. We also made use of the built-in excitation and emission polarisers to obtain isotropic spectra at a magic angle setting. Typical integration time is 0.5 seconds per excitation wavelength, adjusted on a sample-to-sample basis.

When recording SAFE spectra, we used dye solutions with typcial concentrations of a few μM. In general, these concentrations were adjusted so that the total decadic absorbance (A) was less then 0.15. We pipetted typ. 3.5 ml of these solutions into chemically resistant Thorlabs CV10Q35FAE fused silica cuvettes. During measurements, these cuvettes were placed in the SampleSnap4-Pelt temperature-controlled sample holder set at 22 °C. In order to ensure that a continuous supply of fresh molecules, we also used the included magnetic stirrer with a small stirring bead at a stirring speed of 2000 rotations-per-minute (RPM). We found, however, that the actual speed of stirring does not impact the any of the fluorescence spectra we obtained, indicating that dye bleaching is not common.

After measurements, we cleaned the exterior of the cuvettes using ethanol, following the 'Drop and drag method'[4] recommended by Thorlabs. To clean them internally, we rinsed with millipore water and ethanol, followed by drying by nitrogen blow.

In order to process our raw data, we used a number of built-in EzSpec procedures. First, we opted to perform 'background subtraction', based on measurements from solvent-filled cuvettes. Using this process, we could remove signals from cuvette absorption, external reflections, and solvent Raman spectra. As an additional step, we also enabled inner-filtering corrections (IF) of the first and second kind. Using this setting, we compensated light loss in the excitation and emission paths due to solute absorption.[5]

As a result of the thorough calibration performed by HORIBA, the recorded emission spectra are relative wavelength-based spectral irradiance spectra: $I_\lambda^{rel} \propto t_{int}^{-1} \cdot \frac{\partial E}{\partial \lambda}$ with E denoting sampled energy and t_{int} denoting the integration time. We then convert these spectra to energy-based relative photon emission density spectra: $\rho_\varepsilon^{rel} = \frac{I_\lambda^{rel}}{\varepsilon^3} \cdot n(\varepsilon)^2$. Here, the additional n^2 correction term corrects for the solid angle decrease of light collection that happens with increasing solvent refractive index n.

Excitation calibration of EEM measurements in water and ethanol

In the article, we report emission probability density spectra ($\frac{\partial \Phi_f}{\partial \varepsilon_{em}}$) instead of relative photon density spectra (ρ_ε^{rel}). The two are proportional to each other, and the proportionaly factor can be measured via device calibration against dyes with known quantum yields (Φ_f).[1,2,6,7] To measure this factor at a wide range of excitation wavelengths, we devised a calibration procedure applicable for water- and ethanol-based samples.

As a first step, we first determined the device-specific quantum yields (Φ_f^{device}) of the yield standards. In case of IF-corrected SAFE measurements, the device-based yield is calculated as the ratio total photon count rate and the sample absorption: $\Phi_f^{device} = \int \rho_\varepsilon \left(\varepsilon_{exc}, \varepsilon_{em}\right) d\varepsilon_{em} \cdot A^{-1}(\varepsilon_{exc})$.

As a next step, we used our selected dye standards (see Table 3) as well as two extra dyes (DPTS, PTS^-), to prepared dye solutions. After performing a SAFE recording, we replaced $\approx \dfrac{1}{3}$ of the solution with a neat solvent. We repeated this step 4 times, resulting in a concentration series of each selected dye. Using this approach, we found that for all presented dyes behave ideally, as the shapes of their absorption and emission spectra as well as the magnitude of their emission yields were unchanged by concentration. We then processed this concentration series to extract absorption and emission signatures with an enhanced signal-to-noise ratio, see Numerical methods.

Table 3: Reference dyes used in quantum yield measurements.

Dye	Solution	Exc. region [eV]	Reference	Nominal Φ_f
Rhodamine 6G	Ethanol	2.25. . . 2.6	[3,6]	0.94
Fluorescein	0.1M NaOH	2.35. . . 2.7	[6]	0.92
2,7 Dichloro-fluorescein	0.1M NaOH	2.4. . . 2.8	[3]	0.89
Coumarine 153	Ethanol	2.6. . . 3.5	[3,6]	0.53
Quinine hemisulfate	0.105 M $HClO_4$	3.25. . . 3.9	[1,3,6]	0.6

We then proceed with the broadband calibration procedure. In the first step, we compared the collected excitation-dependent device-specific yields of every dye, derived from their first excited states. We illustrated our results in Figure 2 (a). We found, that these device-specific yield spectra follow identical trends regardless of the dye itself. By finding their best overlap and averaging it (see Numerical methods), we obtained the Duetta's excitation sensitivity curve, see Figure 2 (b).

Employing the inverse of that as a correction, we obtained an excellent correlation between the observed and the literature-reported quantum yields of the reference dyes, see Figure 3. Using the slope (K) of this fitted line, we then could calculate the photon emission density spectra in the article: $\dfrac{\partial \Phi_f}{\partial \varepsilon_{em}} = \rho_\varepsilon(\varepsilon_{em}) \cdot K^{-1}$.

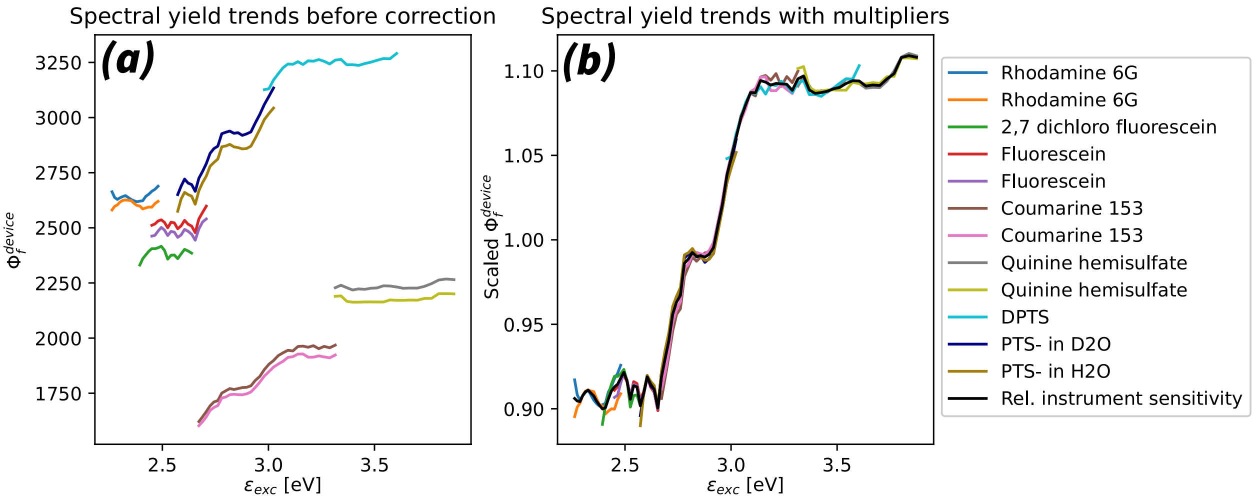

Figure 2: Trends of spectral sensitivity with different dyes. (a) Spectral fluorescence yields (Φ_f^{device}) as measured in device units, plotted in function of excitation photon energy (ε_{exc}). (b) The same yields, with best-match multipliers applied. Black line denotes the thus-obtained excitation sensitivity curve. Dyes appearing twice indicate repeated experiments.

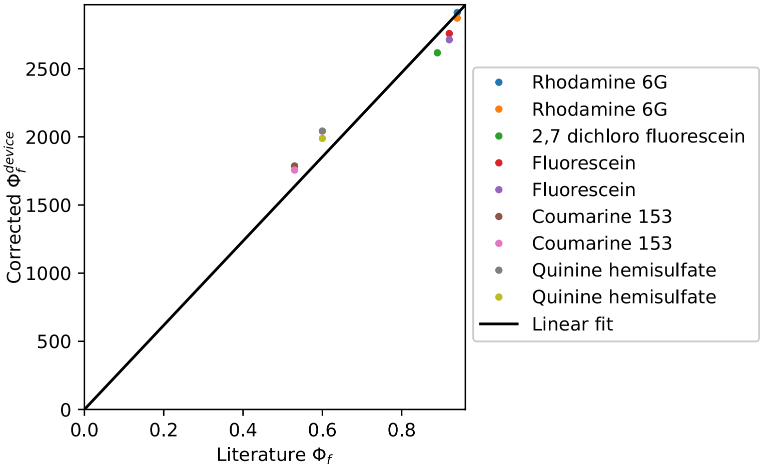

Figure 3: Instrument yield calibration, (a) without sensitivity correction and (b) with sensitivity correction applied. Here, device-specific yields are plotted against literature-reported yield values (Φ_f). Black lines denote best fit through zero in either scenario.

EEM characterisation of AQ^- in H_2O and D_2O

After calibration, we obtained SAFE recordings of H/DAQ and AQ^- in H/D_2O using the same procedures as for yield standards. We obtained H/DAQ solutions were obtained by diluting AQ^- solutions with aqueous solution of $H/DClO_4$. We decided to use perchloric acid instead of hydrochloric acid to prevent fluorescence quenching by chloride ions.[8]

Because we used neat H/D_2O for the AQ^- recordings, we observed that 1...2% of the molecules are in the H/DAQ form. For presentation purposes, we subtracted the H/DAQ absorption and emission spectra. We did that by scaling on the $[3\,eV < \varepsilon_{exc} < 3.35\,eV]$; $[2.1\,eV < \varepsilon_{em} < 2.9\,eV]$ region of the EEM, a region unique to H/DAQ. A detailed description of this procedure is provided in the Numerical methods section.

By analysing the retrieved EEMs, we found that they could be described as a product of an excitation and emission spectrum. We obtained these spectra using a purpose-developed algorithm, see 'EEM decomposition' in Numerical methods. The retrieved emission spectra - obtained by 3.63 eV (342 nm) excitation - are illustrated in Figure 5. We then simultaneously fit all obtained AQ^{-*} and H/DAQ^* emission spectra using just two lognormal bands[9,10] (see Numerical methods). The band parameters are described in Table 4.

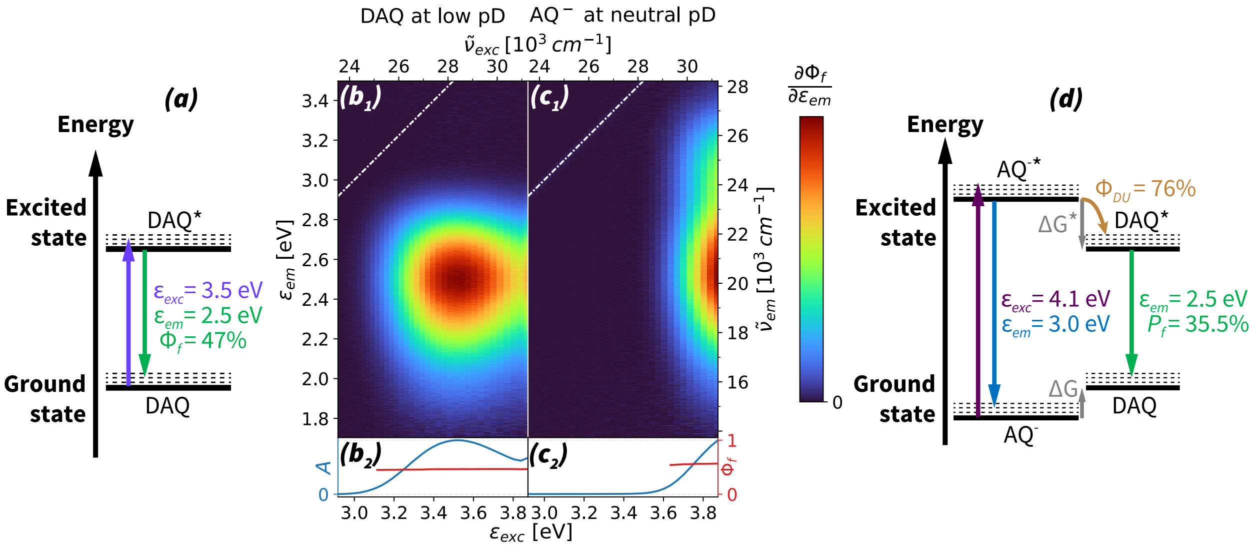

Figure 4: Fluorescent characterisation of AQ^- and DAQ in D_2O.
(b_1-b_2): SAFE recordings of DAQ in acidified D_2O. (a) Retrieved Jablonski-diagram of DAQ. (c_1-c_2): SAFE recordings of AQ^- recorded in neat D_2O. (d) Jablonski-diagram of AQ^-, including proton uptake from D_2O.

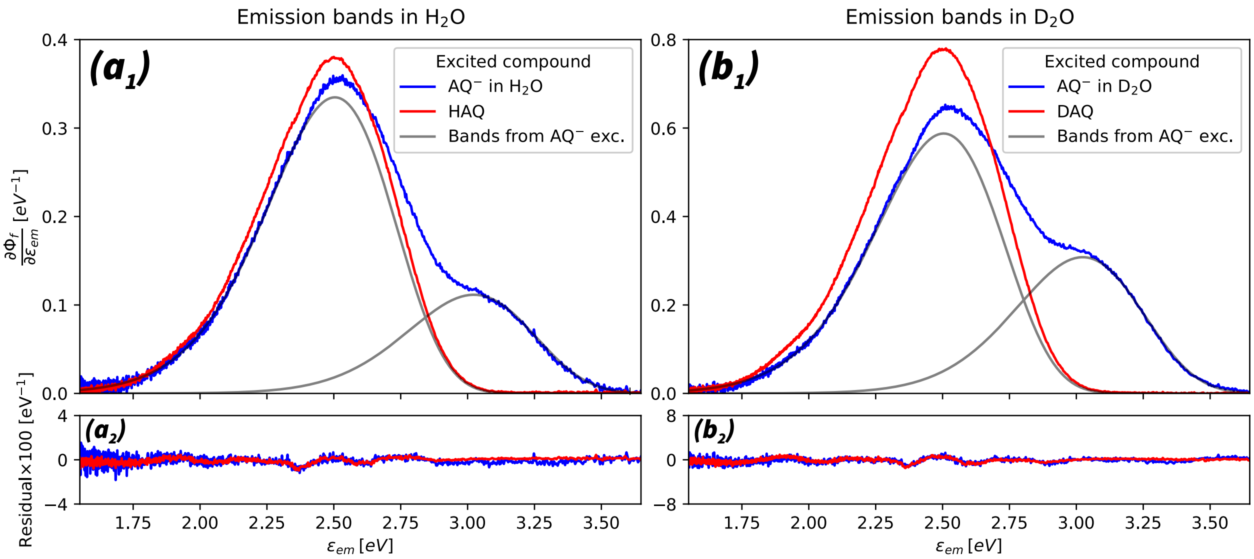

Figure 5: Bandshape analysis of emission probability density ($\frac{\partial \Phi_f}{\partial \varepsilon_{em}}$) spectra following the excitation of (a_1) HAQ and AQ^- in H_2O, and (b_1) DAQ and AQ^- in D_2O. Spectra are plotted against emission photon energy (ε_{em}). Gray, decomposed bands represent best-fitting log-normal bands. Residuals following lognormal band subtraction are presented in (a_2) and (b_2).

Table 4: Band-shape parameters describing best fit attempts on the emission spectra presented in SI Figure 5. We additionally present these after conversion to wavelengths (λ) and wavenumbers ($\tilde{\nu}$).

Parameter	H/DAQ*	AQ^{-*}	Unit
ε_0	2.506	3.020	eV
λ_0	495	411	nm
$\tilde{\nu}_0$	20200	24400	cm^{-1}
$\Delta\varepsilon$	0.560	0.559	eV
$\Delta\tilde{\nu}$	4520	4510	cm^{-1}
$asym.$	-0.217	-0.171	-

Numerical methods

Scaled subtraction

In order to subtract the spectrum $A(\varepsilon)$ from $B(\varepsilon)$ in the region $(\varepsilon_1, \varepsilon_2)$, we use numeric methods to find the scaling factor s that minimises the summed residual squares $SRSQ$: $SRSQ = \int_{\varepsilon_1}^{\varepsilon_2} (B(\varepsilon) - s \cdot A(\varepsilon))^2 \, d\varepsilon = min$. This method is extendable for multi-dimensional spectra.

Concentration series decomposition

In a concentration series of spectra $S(c^i, \varepsilon)$ with unknown concentrations c^i we attempt to extract both the corresponding signature and the individual (relative) concentrations. We thus seek the scaling factors s_i that are proportional to c^i, to minimize the following residual: $SRSQ = \sum_i \int_{\varepsilon_1}^{\varepsilon_2} (S(c^i, \varepsilon) - s_i \cdot S_0(\varepsilon))^2 \, d\varepsilon = min$ where $S_0(\varepsilon)$ is the desired spectrum.

To solve this, we take an iterative approach. In each iteration, we extract S_0 for a fixed set of s_i. For this, we use Python's Numpy.linalg.lstsq() algorithm to retrieve $S_0(\{s_i\})$.

Next, iterating through a sets of s_i is then used to minimize $SRSQ$. For this, we use Python's Scipy.optimize.minimize function with the constraint that $s_0 = 1$. We find that this method reliably extracts both the absorption and EEM signatures with greatly reduced noise.

Excitation sensitivity calculation

During the excitation sensitivity calibration process, obtained a set of curves $\Phi_f^{device,X}$, with X iterating through the relevant compounds. To find their best overlap, we try to minimise the following quantity by adjusting the scaling factors s_i: $SDSQ = \sum_{\varepsilon_{exc}} STD^2 \left(\sum_X s_X \cdot \Phi_f^{device,X}(\varepsilon_{exc}) \right)$ with STD representing the standard deviation of the curves at a given excitation energy, and with the constraint that $s_{i=i_{max}} = 1$. After this calculation, we normalised all s_i with a common scaling factor for presentation purposes, and plotted the matched spectra in Figure 2 (b).

EEM decomposition of a single excited state

This method is used on dyes that possess an excitation-independent emission and quantum-yield spectrum. Here, the numeric hypothesis is that $\rho_\varepsilon(\varepsilon_{em}, \varepsilon_{exc}) = P(\varepsilon_{em}) \cdot Q(\varepsilon_{exc})$. Here, $P(\varepsilon_{em})$ is the extracted emission profile, and $Q(\varepsilon_{exc})$ is the extracted attenuation profile. To obtain these, we use the same algorithm as for a concentration series, except we replace s_i with $Q(\varepsilon_{exc})$. In general, the resulting Q and P have a scaling ambiguity, which we can easily resolve. Because we observe a single excited state, $Q(\varepsilon_{exc}) \propto A(\varepsilon_{exc})$. As such, we scale Q to match A, and perform the inverse scaling on P. Combined with instrument calibration, we calculate the photon emission probability density spectra.

Emission band fitting

When performing band fitting,[9,10] we used log-normal bands to find the best fit parameters. The functions describing band-shapes have the following mathematical shape: $S(\varepsilon, \varepsilon_0, \Delta\varepsilon, asym.) = 2^{-\left(\frac{ln\left(1 + 2 \cdot \frac{asym. \cdot (\varepsilon - \varepsilon_0)}{\Delta\varepsilon}\right)}{asym.} \right)^2}$. Here, ε_0 represents the band centre, $\Delta\varepsilon$ represents the band width, and $asym.$ represents an asymmetry factor of this band.

We used these bands to simultaneously fit the H/DAQ*, and AQ^{-*} emission signatures in both solvents. We minimised the following sum: $SRSQ = \sum_{\varepsilon_{em}} \left(\rho_\varepsilon^{HAQ^*}(\varepsilon_{em}) - a_1 \cdot S_A\right)^2 + \left(\rho_\varepsilon^{DAQ^*}(\varepsilon_{em}) - a_2 \cdot S_A\right)^2 + \left(\rho_\varepsilon^{AQ^{-*}_{H_2O}}(\varepsilon_{em}) - a_3 \cdot S_A - b_3 \cdot S_B\right)^2 + \left(\rho_\varepsilon^{AQ^{-*}_{D_2O}}(\varepsilon_{em}) - a_4 \cdot S_A - b_4 \cdot S_B\right)^2$ with $\rho_\varepsilon = \frac{\partial \Phi_f}{\partial \varepsilon_{em}}$ representing

emission probability density, a_i, b_i being scaling factors for acidic and basic emissions, and S_A and S_B representing the corresponding acidic and basic emission signatures. In each iteration with a_i and b_i, we recovered S_A and S_B the same way as for the concentration series, and then used these to calculate $SRSQ$.

UV-VIS measurements

Experimental methods

Quantitative UV-VIS spectra between 200 and 700 nm were recorded by using a Perkin-Elmer Lambda 35 spectrometer, using 1 nm slit width and step size. Each presented spectrum is an average of 3 consecutive recordings. Using these settings, we measured the spectrum of liquid samples in a Starna Ultra-Micro 100 μm fixed thickness UV silica flowcell. After each measurement, we cleaned the sample cell similarly to the quartz cuvettes in the fluorescence measurements.

Before measuring samples, we recorded background spectra with the sample cell removed. Next, we measured the spectrum of a sample cell filled with neat water. We then proceeded to measure the absorption spectrum of desired solutions, and subtracted the absorption of the solvent-filled cell. This way, we removed the absorption and reflection signals from the cuvette, both from its contents and from its surfaces.

To provide quantitative results, we measured its thickness (d) by analysing the fringe spacing in the transmission spectrum of the empty cell.[11] Using this method, we obtained that $d = 111.2 \pm 3.5 \mu$m. We used this value to calculate decadic molar attenuation coefficients (ϵ_{att}) from the measured absorption spectra.

UV absorption spectra of AQ^-, HAQ and HSI

We determined the decadic molar attenuation coefficients of AQ^-, by measuring absorption of pH adjusted solutions of 10 mM AQ^- and 3 mM HAQ in H_2O. We performed similar measurements for 100 mM of HSI as well. We compare these to our recordings from the Duetta, as seen in Figure 6. Here, we scaled the spectra from the Duetta, so that they match those from the the Perkin-Elmer spectrometer (see Numerical methods of this section). This way, we could determine respective component concentrations in spectra measured by the Duetta. Comparing recordings from the two devices, we find no meaningful differences between the absorption spectra at a few μM concentrations from the Duetta and those at a few mM concentrations from the Perkin-Elmer, which indicates ideal dye behaviour without aggregation.

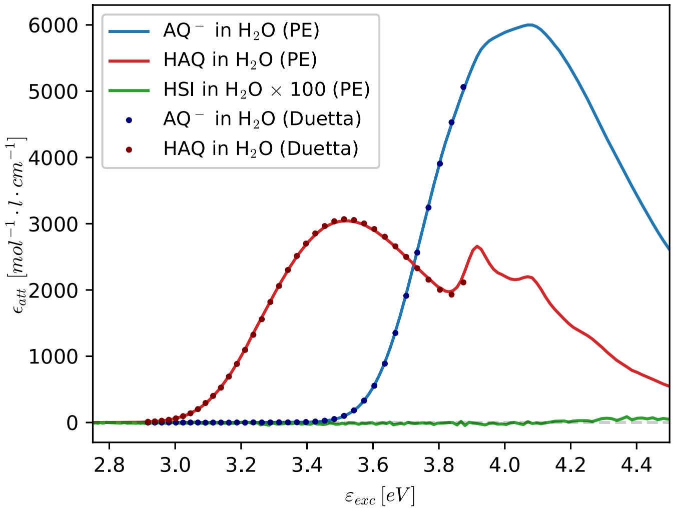

Figure 6: Solid lines: Decadic molar absorption coefficient spectra ϵ_{att} obtained for AQ^-, HAQ and HSI recorded with the Perkin-Elmer spectrometer (PE), blueshifted by 4.7 meV. HSI attenuation has been scaled 100× for presentation purposes. Dots: UV-VIS spectra of AQ^- and HAQ obtained with the Duetta, scaled to match the IRF-adjusted spectra from the Perkin-Elmer spectrometer.

Table 5: Parameters describing the maximum of absorption spectra of AQ^- and HAQ, used in our Förster-cycle analysis.

Parameter	H/DAQ*	AQ^{-*}	Unit
ε_{max}	3.512	4.065	eV
λ_{max}	353	305	nm
$\tilde{\nu}_{max}$	28300	32800	cm^{-1}

Ground-state acidity of HAQ

In an attempt to determine the acid-base dissociation constant (K_a) of HAQ, we performed a combined absorption and emission (SAFE) measurements alongside titration. We then perform quantitative analysis on the absorption spectra, and obtain K_a.

We first created μM HAQ solutions by diluting AQ^- solutions in 1 M $HClO_4$ solution. In each step, we first measured the absorption of the solution. Next, we displaced $\approx \frac{2}{3}$ of the solution, and replaced it with millipore water. We also replenished the dye content by adding some more of the AQ^- solution. We then measured the solution pH using a Mettler-Toledo FiveEasy pH meter. We performed these steps several times, each one resulting in a slightly increased pH value. We found that above pH 0.69, spectral properties of HAQ/HAQ* do not vary with pH, with only minor, likely ionic shifts below this pH. We have to note that at pH values between 6 and 8, our pH meter is less accurate and more prone to fluctuations, mainly due to the low ionic strength in the solutions we used.

We then also repeated this process starting from a pH of 11.7, decreasing the pH after each step. We did this because we found that at pH values higher than 11.7, the UV illumination of the Duetta slowly degrades AQ^- in solutions. To confirm that AQ^- spectra starting from this pH value are unaffected, we compared them to those that started from pH 10. We found them to be identical, showing no sign of degradation.

Having obtained quantitative information about the absorption spectra of both AQ^- and HAQ, we analysed this spectra to obtain concentrations of both species at each pH values (see Numerical methods). We then calculated the ratios of the two in Figure 7. We determined the ground-state acidic strength ($pK_a = -log_{10}(K_a)$) of HAQ by fitting this ratio obtained from the Henderson–Hasselbalch equation: $\frac{c^{AQ^-}}{c^{AQ^-} + c^{HAQ}} = \frac{10^{pH-pK_a}}{1 + 10^{pH-pK_a}}$.

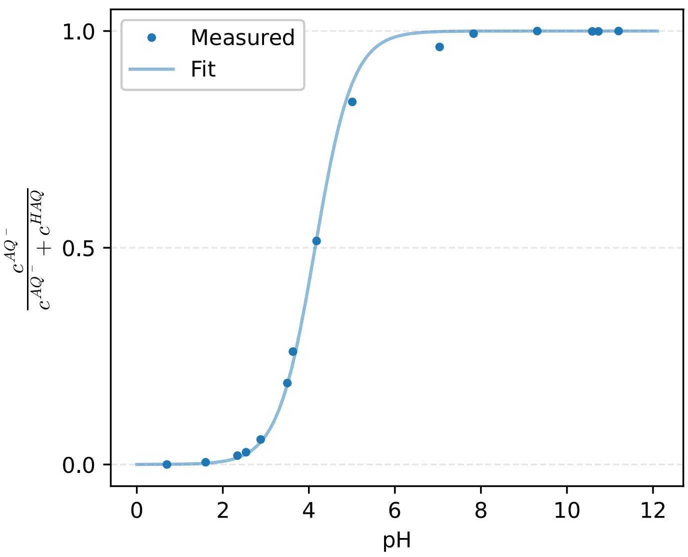

Figure 7: Molar ratio of AQ^- at different pHs, derived from quantitative absorption measurements. The solid line represents the best fit through these points.

Association properties of actinoquinol and succinimide

We prepared a series of pH-adjusted AQ^- - succinimide solutions in H/D_2O, with 10mM AQ^- concentration and 0 to 2 M succinimide concentrations. We primarily used these samples to execute the transient absorption measurements, in addition to which we also measured their UV absorption spectra several days later.

We note, that during this time, the samples underwent slight acidification, likely due to ambient CO_2 absorption. This means that HAQ content was increased from 0 to 2.5 %, calculated based on UV-VIS spectra. As such, we routinely subtract H/DAQ absorption by scaling onto the [3 eV< ε_{exc} <3.35 eV] spectral region, which is unique to H/DAQ. After this, we also correct the concentration of AQ^- inside the solution. As a result, we obtained the absorption series purely corresponding to AQ^-, as seen in Figure 8.

In the main text, we propose that this redshift is due to direct hydrogen bonding between succinimide and actinoquinol molecules. This association process would then be governed with a single equilibrium constant: $K^{assoc} = \frac{c^{AQ^- \cdots HSI}}{c^{free\ AQ^-} \cdot c^{HSI}}$. We seek to determine this constant using the UV-VIS measurements. We describe a corresponding procedure in the Numerical methods section, and display the corresponding parametric fits in Figure 9.

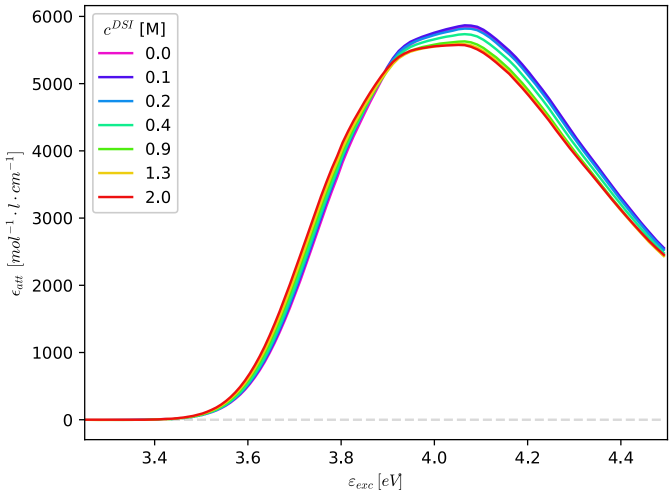

Figure 8: Red-shift of AQ^- absorption, with increasing succinimide concentrations in D_2O. Note, that the redshift cannot originate from DAQ, because any DAQ absorption (<2.5 % of AQ^- content) were subtracted before calculating attenuation coefficients.

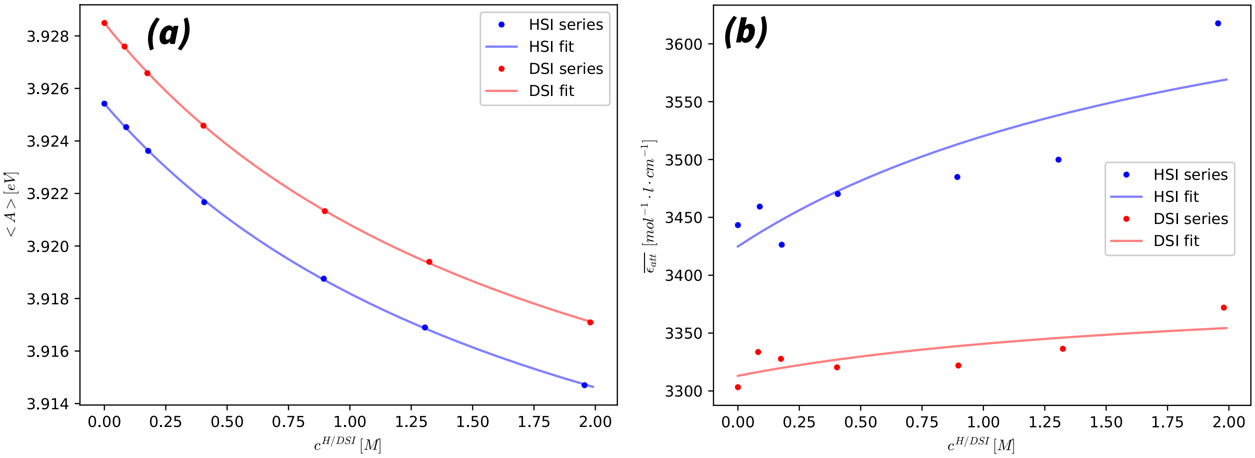

Figure 9: Curve fits for determining the association constant of succinimide and AQ^-. (a) Fit for experimentally obtained first-order-absorption moments ($\langle A \rangle$). (b) Fit for experimentally obtained average absorption coefficients ($\overline{\epsilon_{att}}$).

Numerical methods

Matching spectra between the Perkin-Elmer spectrometer and the Duetta

We made Duetta and Perkin-Elmer (PE) spectra comparable convolving we convolved the PE spectra with a Gaussian function with a 3 nm full-width half-maximum (FWHM), to match Duetta settings. We also applied a manual offset of 4.7 meV to the PE spectra, so that they better match the wavelength calibration of the Duetta. In the final step, we interpolated the PE spectra to the wavelengths of the Duetta recording, to which we then scaled the Duetta recordings.

Analysing acid-base titration spectra

In analysing spectra recorded at different pHs, we found that spectra between pH 0.7 and pH 12 can be accurately described as a linear combination of just two spectra, corresponding to HAQ and AQ^-.

To decompose the absorption spectra, we then minimized the following sum: $SRSQ = \sum_{pH} \left(A_{exp}(\varepsilon_{exc}, pH) - s_{HAQ}(pH) \cdot S_{HAQ}(\varepsilon_{exc}) - s_{AQ^-}(pH) \cdot S_{AQ^-}(\varepsilon_{exc}) \right)^2$, with the constraints that $s_{HAQ}(pH = 0.7) = 1$, $s_{AQ^-}(pH = 0.7) = 0$ and that $s_{HAQ}(pH = 11.7) = 0$, $s_{AQ^-}(pH = 11.7) = 1$. Similarly to the concentration-decomposition in the fluorescence section, in each step we took a set of scaling factors ($s_i(pH)$), calculated the best fitting spectral signatures ($S_i(\varepsilon_{exc})$), and finally the $SRSQ$ value. By iterating on the scaling factors, the obtained $SRSQ$ values converged to a minimum. By scaling the obtained AQ^- and HAQ signatures onto the measurements from the Perkin-Elmer spectrometer, we then calculated the individual concentrations of AQ^- and HAQ at each pH values.

Analysis of actinoquinol absorption redshift

By combining the association equlibrium equation ($K^{assoc} = \dfrac{c^{AQ^- \cdots H/DSI}}{c^{freeAQ^-} \cdot c^{H/DSI}}$), and conservation of mass ($c^0 = c^{freeAQ^-} + c^{AQ^- \cdots H/DSI}$), we can calculate the ratio (r^{assoc}) of the associated molecules: $r^{assoc} = \dfrac{K^{assoc} \cdot c^{H/DSI}}{1 + K^{assoc} \cdot c^{H/DSI}}$. Here, we used the approximation that the succinimide concentration is constant regardless of how much AQ^- molecules are associated. This is justified since $c^{H/DSI} >> c^{AQ^-}$.

Our primary assumption is that a linear combination of free and associated AQ^- molecules describe the observed spectra: $\epsilon_{att}(\varepsilon_{exc}, c^{H/DSI}) = ((1 - r^{assoc}(c^{H/DSI})) \cdot \epsilon^{free}(\varepsilon_{exc}) + r^{assoc}(c^{H/DSI}) \cdot \epsilon^{assoc}(\varepsilon_{exc}))$. We chose fit the first order moment of the absorption spectra in the 3.5...4.15 eV region, which describes the red-shift of the absorption: $< \epsilon_{att} > (c^{H/DSI}) =$
$= \dfrac{\int_{3.5\,eV}^{4.15\,eV} \epsilon_{att}(\varepsilon_{exc}, c^{H/DSI}) \cdot \varepsilon_{exc}\, d\varepsilon_{exc}}{\int_{3.5\,eV}^{4.15\,eV} \epsilon_{att}(\varepsilon_{exc}, c^{H/DSI})\, d\varepsilon_{exc}} = \dfrac{< \epsilon^{free} > + < \epsilon^{assoc} > \cdot K^{assoc} \cdot b \cdot c^{H/DSI}}{1 + K^{assoc} \cdot b \cdot c^{H/DSI}}$. Here, b is the ratio of average attenuation coefficients in this window: $b = \dfrac{\overline{\epsilon^{assoc}}}{\overline{\epsilon^{free}}}$. By fitting the redshift, we obtain ($K^{assoc} \cdot b$).

To obtain K^{assoc} on is own, we need to additionally fit the averaged attenuation as well: $\overline{\epsilon_{att}}(c^{H/DSI}) = \frac{\int_{3.5\ eV}^{4.15\ eV} \epsilon_{att}(\varepsilon_{exc}, c^{H/DSI}) \cdot \ d\varepsilon_{exc}}{4.15\ eV - 3.5\ eV} = \left(1 + (K^{assoc} \cdot b) \cdot c^{H/DSI}\right) \cdot \frac{\overline{\epsilon^{free}}}{1 + K^{assoc} \cdot c^{H/DSI}}$. Having obtained $(K^{assoc} \cdot b)$ from the previous fit, we can directly include it here. As such, the second fit will directly yield K^{assoc}.

Infrared absorption measurements

Experimental methods

Infrared absorption spectra were recorded using the Bruker Vertex 80v Fourier-transform spectrometer with a resolution of 1 cm^{-1}. We placed liquid samples between two, 25.4 mm diameter, 0.5 mm thick UV-grade CaF_2 windows (Crystran CAFP25.4-0.5U). These windows were separated by 25 micron fluorinated ethylene propylene (FEP) spacers, or 50 micron thick polytetrafluoroethylene (PTFE) spacers.

We executed the absorption measurements under nitrogen atmosphere, to prevent ambient absorption of CO_2 and H_2O in the air. We started with a background measurement, where no sample cell was placed in the beampath. Next, we recorded and averaged the absorption spectrum based on 50 scans of the interferometer. After measurements, sample cells assemblies were disassembled, and every optical surface was cleaned with the 'Drop and drag' method,[4] using water and ethanol.

Assignment of main AQ^-, HAQ and DAQ features

In Figure 4 (a) of the main text and SI Figure 13 (a), we present the absorption of AQ^-, HAQ and DAQ with the solvent background subtracted (see Numerical Methods). We proceed to assign their strongest observable features, starting with HAQ and DAQ.

By comparing HAQ and DAQ features, we find that their strongest features appear at different frequencies, at 1560 cm^{-1} and at 1535 cm^{-1}. Given that the two species are otherwise electronically identical, we infer that this frequency difference is due to the difference in the mass of the attached hydrogen and deuteron. As both modes lie in a frequency region typical to aromatic C=C stretching modes,[12,13] we tentatively assign them to ring modes of H/DAQ, which include the NH/D group as well.

In case of AQ^-, removing the proton/deuteron from HAQ/DAQ changes the electronic structure. When compared to H/DAQ, we observe that there are no observable modes at 1535 or 1560 cm^{-1} in H_2O and D_2O, but there are two different modes at 1507 and 1575 cm^{-1}. Because of their characteristic frequencies and the proximity of now-assigned H/DAQ features, we propose that these two modes too correspond to ring modes of AQ^-.

Numerical methods

Solvent background subtraction

Like for UV-VIS measurements, we also developed a procedure to remove the window reflections, as well as absorption of CaF_2 and the solvent components. We do this by separately determining their respective

absorption spectra, and then performing a scaled subtraction on them. Our approach is presented in Figure 10, and a similar approach is presented in an earlier work.[14]

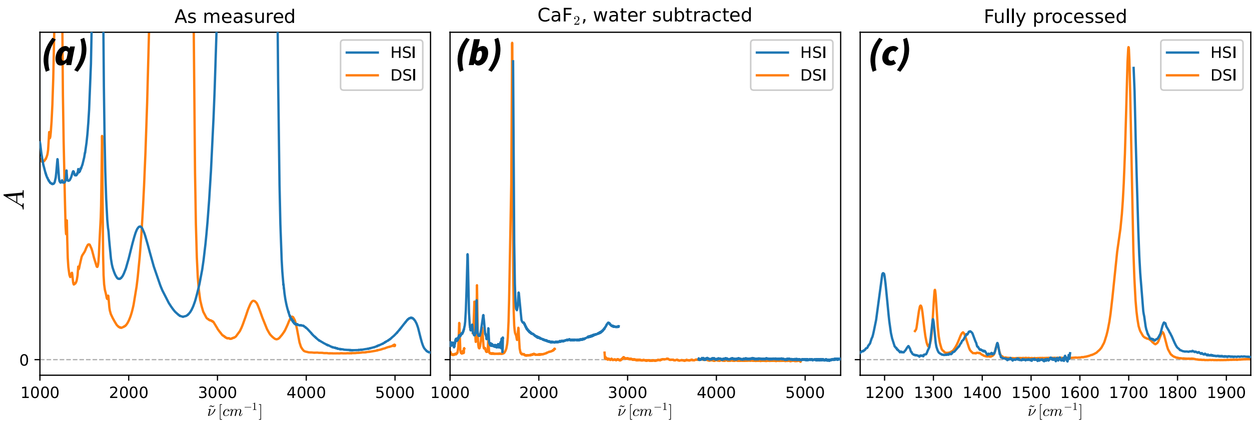

Figure 10: Absorption (A) of H/DSI at various steps of processing, plotted in function of spatial frequency ($\tilde{\nu}$). (a) As measured. (b) With CaF_2 and water absorption removed. (c) Fully processed, after removing residual H/DCl absorption.

We first determined the contributions of 1 mm CaF_2 inside the sample cells, by based on the measurements of 0.5mm and 2 mm windows. We remove this from every spectra we measure.

As a next step, we determined the absorption of H_2O, 1 M NaOH in H_2O and 2 M HCl in H_2O, after the CaF_2 contributions were removed. We separated contributions from water and hydrated HCl/NaOH by normalising and subtracting the water spectrum on the combination band of OH stretching and bending.[11]

We used these to determine the spectra from the absorption AQ^- and HSI. After recording absorption of pH-adjusted 100 mM solutions, we first removed CaF_2 and water signals. We followed up with removing contributions from hydrated HCl/NaOH as well. We do this by normalising and subtracting their absorption in a manually selected region where AQ^- or HSI do not absorb, between 1900. . . 2800 cm^{-1}. The accuracy of this process is illustrated by the flat baselines in Figure 10 (c).

For D_2O, we proceed very similarly. We separately determine D_2O and HOD absorption, as well as absorption of hydrated DCl and NaOD. The main difference is that we use different frequency regions normalisation.[11] The resulting spectrum of DSI is plotted in Figure 10 (c).

In case of SI^-, we need to take additional steps. Unfortunately, we cannot directly observe its infrared features[15] as it slowly hydrolyses at high pHs over longer timescales.[16] According to our observations, it can co-exist with HSI while both remain stable. We thus measured the absorption spectrum of solutions with mixed H/DSI and SI^- content with a total concentration of 100 mM. After removing solvent and window contributions, we also removed the HSI features by normalising it using the C=O stretching region around 1700 cm^{-1}.

In case of HAQ and DAQ, we also needed extra steps. The primary reason is that they have low solubility of a few mM, which significantly decreases the signal-to-noise ratio. To resolve this, we averaged HAQ and H_2O absorption over 500 scans instead of just 50. Following the window and solvent removal procedure, the remaining HAQ and DAQ features remained on top of a mildly structured background as their observed spectral features are comparably sized to the error of the subtraction process. As such, we applied additional offsets for presentation purposes.

Transient absorption measurements

Experimental methods

We recorded transient absorption (TA) spectra using a home-built experimental setup. In this setup, we use ultrashort UV pump and mid-infrared probe pulses centered around 342 nm (3.63 eV, 29240 cm^{-1}) and near 6.66 μm (0.186 eV, 1500 cm^{-1}), to start and observe chemical reactions in liquid samples. We offer a schematic in Figure 11.

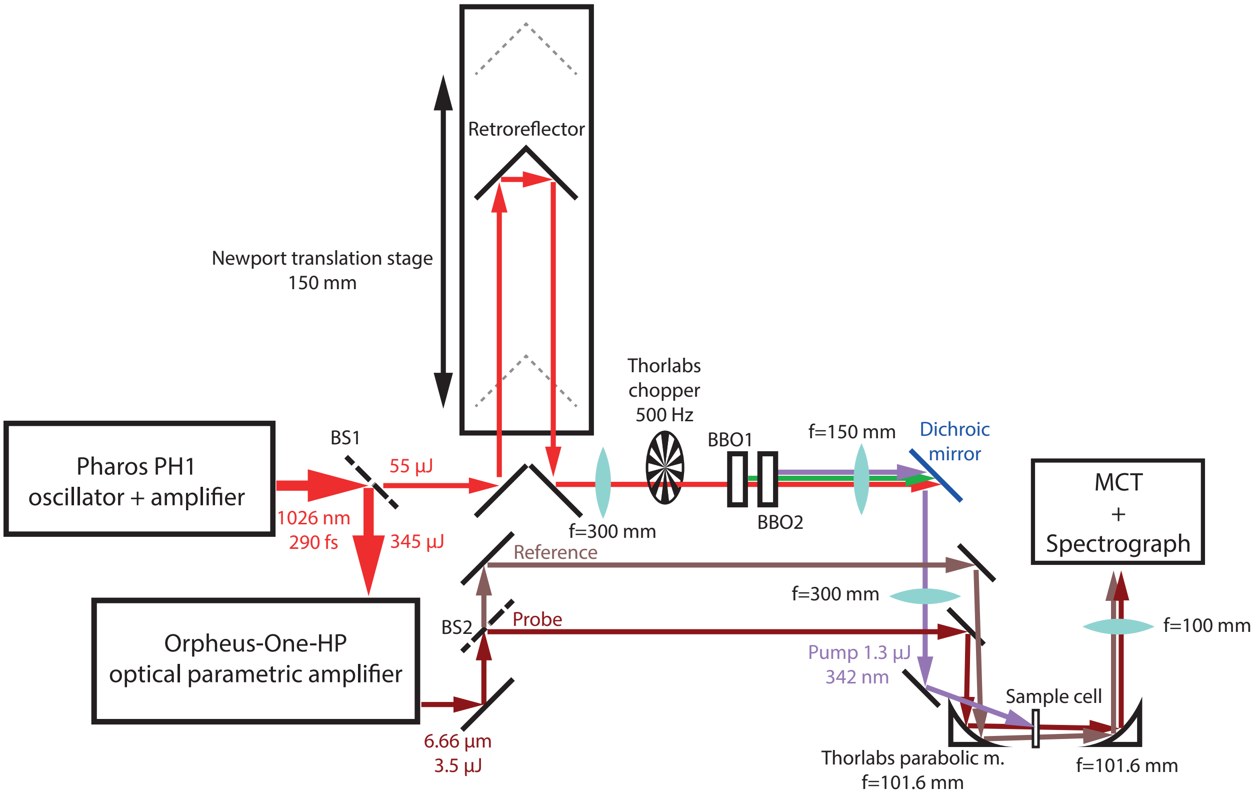

Figure 11: Illustration of the light generation and guidance elements in the transient absorption experimental setup. Black lines denote mirrors, dashed line denote beamsplitters (BS) and the arrows denote the direction of laser beams, and cyan shapes denote convex lenses with the corresponding focal lengths.

We generate near-infrared pulses using a Light-Conversion Pharos PH1 oscillator-amplifier system at a 1 kHz repetition rate, with a pulse energy of 400 μJ. These pulses are centered around 1026 nm, with a time-duration of 300 fs. We split these to separately generate the pump and probe pulses.

In the pump-arm, we delay the fundamental 1026 nm pulses using a retro-reflector placed on a mechanical translation stage. This stage has a translation range of 150 mm, allowing us to adjust pump-probe delay (τ_{pp}) up to 1 ns. Following delay, we eliminate every second pulse using an optical chopper, yielding a 500 Hz pump rate. We then generate third harmonic at 342 nm using a pair of BBO crystals. After generation,

separate the 342 nm component using a dichroic mirror. We adjust the UV intensity using neutral density filters, obtaining a typical pulse energy of 1.3 μJ. We additionally adjust the beam polarisation using $\frac{\lambda}{2}$ waveplate. As a last step, we use a lens to focus this beam onto a sample cell.

This sample cell is very similar in construction to the FTIR cell, but built smaller. To ensure that after every pump pulse illuminates a fresh spot, we placed our sample cell in a high-speed rotating-cooling assembly, set at 1000 revolutions-per-minute and at 22 °C. Using the knife-edge method, we measured the pump spot size to be $\approx$ 150 μm full-width half maximum (FWHM), which is much smaller than the $\approx$ 600 μm the illuminated spot travels between consecutive pump shots.

To generate mid-infrared probe pulses with tunable frequency, we used a Light-Conversion Orpheus One HP, multi-stage collinear optical parametric amplifier (OPA) with a differential-frequency generation (DFG) extension. On the output, we obtain a typical pulse energy is 3.5 μJ in the 1500 cm^{-1} region. To avoid ambient H_2O absorption from the air, we execute TA experiments under low-humidity conditions ($RH < 8\%$).

After generation, we split the light into probe and reference arms, and separately adjust their intensity using two pairs of wire-grid polarisers. We then directly focus these beams onto the sample cell using off-axis parabolic mirrors. The probe spot has a width of $\approx$ 105 μm FWHM, and overlaps with the pumped spot, while the reference spot does not.

After the sample cell, we sample the probe and reference beam polarisation using a computer-controlled polariser, which we adjust to pump-parallel or pump-perpendicular settings. After recollimation, we focus both beams using a BaF_2 lens, onto the entry slit of a spectrograph. At its output, both beams are dispersed onto two, height-separated, linearised MCT detector arrays (Infrared Associates).

We then collect dark current-corrected light intensity values for every pulse. At each delay point and at each polarisation setting, we typically record intensity values of 5000 pulses in total. As the probe pulse records source fluctuations together with pump-induced signal changes, we use the reference pulses to correct for the source fluctuations.[17] Using this referencing scheme, we could enhance the signal-to-noise ratio of our measurements. We then construct isotropic TA signals by combining the polarisation-resolved signals: $\Delta A_{iso} = \dfrac{\Delta A_{\parallel} + 2 \cdot \Delta A_{\perp}}{3}$.

To correctly identify the temporal overlap of the pump and probe pulses, we separately record the called cross-phase modulation signal (XPM) in solvent-filled cells.[18] By analysing this signal, we can establish the temporal overlap of the pump and probe pulses (τ_{pp}=0) with a <50 fs accuracy.

Transient absorption of AQ- in H_2O and in D_2O

We prepared a series of succinimide solutions in H/D_2O, as described in the UV-VIS section. We recorded their transient absorption spectra in two spectral windows, and stitched them together based on the AQ^- ground-state bleach signal at 1507 cm^{-1}. We also prepared H/DAQ samples with $c^{H/DAQ}$ ≈10 mM.

In transient absorption measurements with AQ^- and H/DSI samples, the observed signals primarily emerge as a result of exciting AQ^-. We do not observe any signals emerging from excited HSI, as its attenuation coefficient is several orders of magnitude smaller, see Figure 6. At pump-probe delays around 0 ps, we observe additional signals from two sources. One source is XPM observed in the CaF_2 windows. This signal is inherent to all transient absorption experiments, and does not contribute to observed signals beyond the temporal overlap of the pump and probe pulses. A second, minor signal is also observed, vanishing on a time-scale of a few hundred fs. This signal attributed to short-lived multi-photon effects[19] attributed to a very small portion of excited molecules. Since we only analyse signals at 1 ps and beyond, we do not need to consider any of the short-lived signal sources before.

We additionally report that our TA signals from the AQ^- samples remained stable during our measurements. One exception is in neat D_2O, where we observe that about <5% of AQ^- ground-state bleach signal is lost over the course of the measurement, which is ≈<2.5% after averaging. Parallel to this, we also observe a rather minor TA signal (<0.05 gsb. units) rising at 1518 cm^{-1} at τ_{pp} > 300 ps, which is not present in DAQ^* measurements. We do not observe such signal degradation effects of AQ^- or SI^- signals in any other samples. As such, we exclude the 1515...1521 cm^{-1} region from our analysis of D_2O-based measurements.

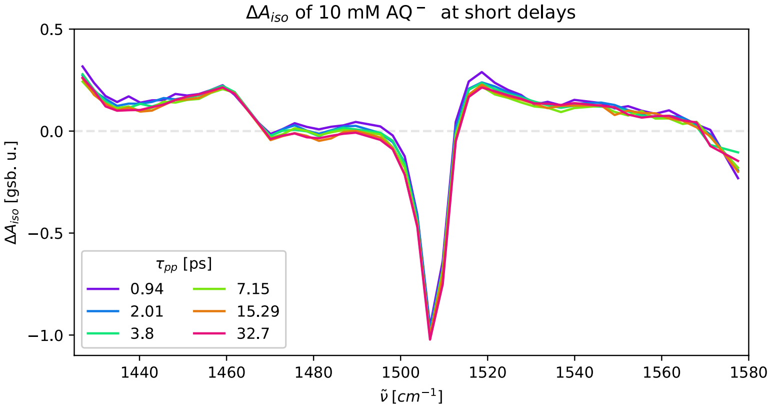

Figure 12: Transient absorption spectra of AQ^- at short delays illustrating subtle changes assigned to solvent relaxation.

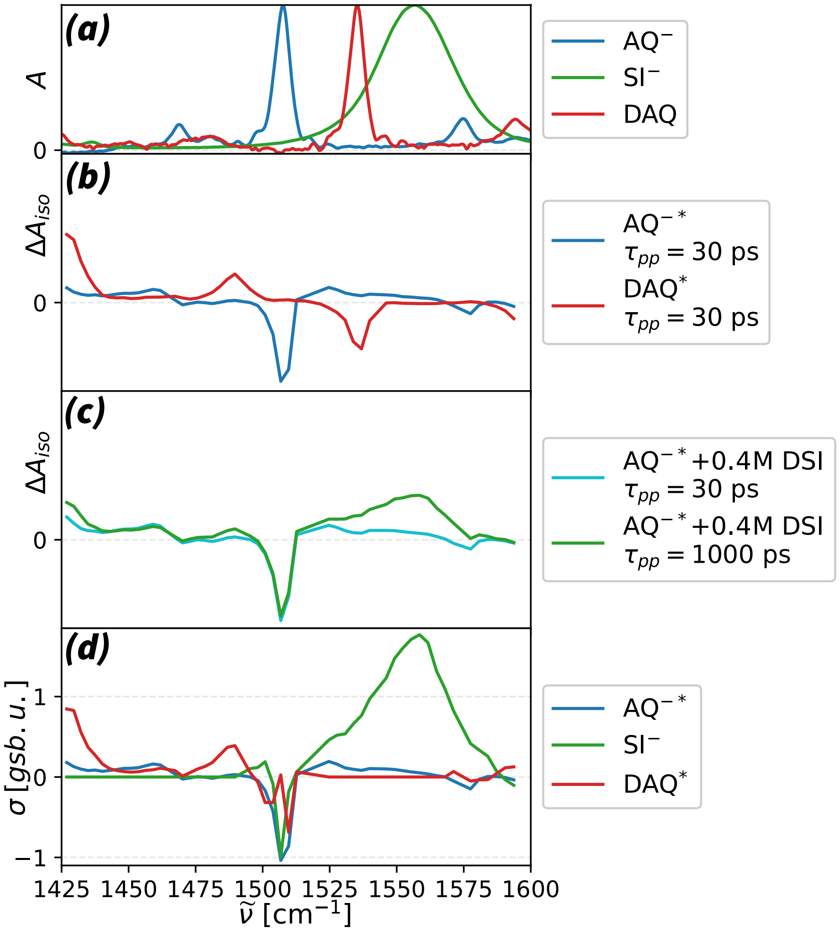

Figure 13: Comparison of the main infrared features in D_2O.
(a) Steady-state infrared absorption spectrum of AQ^-, SI^- and DAQ with solvent-features subtracted. (b) Normalised isotropic transient absorption spectra of key systems at different delays. (c) Transient absorption spectrum of AQ^{-*} in a solution of 0.4 M HSI in in H_2O, at $\tau_{pp} = 30$ ps and at $\tau_{pp}= 1000$ ps, showing emerging DAQ^* and SI^- features. (d) Main component signatures resulting from soft kinetic modelling.

Numerical methods

In this work, we analyse different transient absorption spectra with different approaches. For H/DAQ* samples, we developed a polarisation-resolved extension to kinetic modelling, to retrieve their reorientation dynamics. For H/DSI concentration series, we extract the dynamics the proton transfer using purpose-developed 'soft kinetic modelling'. This method allows us to analyse entire measurement series in one go, and helps separate even the highly correlated dynamics of H/DAQ* and SI$^-$. We then utilise the extracted signatures to best fit our kinetic reaction model describing reaction dynamics.

Kinetic modelling overview

In kinetic modelling, we first establish a mathematical framework based on preliminary understanding of the chemical system being investigated. This involves identifying a number of transient physical-chemical states (A, B, C, D, ...), and constructing a mathematical model to calculate their relative concentration/population over time ($n^A(t)$, $n^B(t), \dots$). These dynamics are typically defined by a set of kinetic parameters, such as $k_{A\to B}$, $k_{A\to C}$, $k_{B\to C}, \dots$. Using these dynamics, one proceeds to extract spectral signatures (σ_A, $\sigma_B, \dots$), by calculating the best fitting values that best replicate the transient absorption dataset ($\Delta A(\tilde{\nu}, \tau_{pp})$). The reconstructed dataset is then compared to the measured one, where the degree of similarity/fit quality (Q) is calculated. To find the best fit, a black-box optimizer algorithms iterates through the kinetic parameters to find the best quality factor ($Q\{k_{A\to B},\ k_{A\to C},\ k_{B\to C}, \dots\}$).

Retrieving spectral signatures

To retrieve best fitting spectral signatures, we use the following quality factor for obtaining the spectral signatures and optimising the fits: $Q = \sum_{\tau_{pp},\tilde{\nu}} \Big(AVG(\Delta A(\tau_{pp}, \tilde{\nu})) - \sum_j n^j(\tau_{pp}) \cdot \sigma^j(\tilde{\nu}) \Big)^2 \cdot w^2(\tau_{pp}, \tilde{\nu})$ with AVG marking the averaged transient absorption over repeated measurements, and $w^{-1} = STD^*(\Delta A(\tau_{pp}, \tilde{\nu})) = \sqrt{STD_0^2 + STD(\Delta A(\tau_{pp}, \tilde{\nu}))}$ marking an adjusted standard deviation based of the repeated measurements. Here, we used the STD_0 value derived from the standard deviation of measurements with blocked pump beams, describing the noise floor of the detection. This term w might include manual weights as well. We can then calculate the spectral signatures for every sampled $\tilde{\nu}$ separately, by minimizing the following factor: $Q = \sum_{\tilde{\nu}} Q_{\tilde{\nu}} = \sum_{\tilde{\nu}} \Big([AVG(\Delta A(\tau_{pp}, \tilde{\nu})) \cdot w(\tau_{pp}, \tilde{\nu})] - \sum_j \big[n^j(\tau_{pp}) \cdot w(\tau_{pp}, \tilde{\nu})\big] \cdot \sigma^j(\tilde{\nu}) \Big)^2$.

Population dynamics using (traditional) kinetic modelling

In the traditional approach, one assumes that a set of first-order reaction constants are sufficient to describe reaction dynamics: $\dfrac{\partial}{\partial t} n^i = -\sum_j k_{ij} \cdot n^j$, with k_{ij} describing rate constants connecting different species. We

reformulate this in a matrix form: $\frac{\partial}{\partial t}\underline{n} = -\underline{\underline{k}} \cdot \underline{n}$, with the initial conditions of $\underline{n}(t=0) = \underline{n^0}$. The solution of this equation is then $\underline{n}(t) = exp\left(-\underline{\underline{k}} \cdot t\right) \cdot \underline{n^0}$. Note, that quantities with a single underscore (e.g. $\underline{u}$) denote vectors, and with a double underscore (e.g. $\underline{\underline{k}}$) denote matrices.

We illustrate the fitting method method with the case of H/DAQ* transient absorption measurements. Because we excite H/DAQ* at 3.63 eV, higher than their absorption maximum of 3.5 eV, they are promoted to an electronically and vibrationally excited state, H/DAQ$^*_{vib}$. These then relax to H/DAQ*, causing a feature-blueshift over a few picoseconds, which was also seen in earlier works.[19] To model the first 300 ps of this process, we use the assumption that all H/DAQ molecules are promoted to the same H/DAQ$^*_{vib}$, and that these molecules relax with the first-order rate constants k_{vib}^{H/DAQ^*} and k_{ES}^{H/DAQ^*}. We also describe this using our formalism: $\begin{bmatrix} n^{H/DAQ^*_{vib}} \\ n^{H/DAQ^*} \end{bmatrix} (\tau_{pp} = 0) = \begin{bmatrix} 1 \\ 0 \end{bmatrix}$ and that $\underline{\underline{k}} = \begin{bmatrix} -k_{vib}^{H/DAQ^*} - k_{ES}^{H/DAQ^*} & 0 \\ k_{vib}^{H/DAQ^*} & -k_{ES}^{H/DAQ^*} \end{bmatrix}$.

Here, we omit the newly relaxed H/DAQ populations as they yield no transient absorption signature once they return to the ground state. Based on our fits, k_{ES}^{H/DAQ^*} >10 ns, which also means that they will have no meaningful contribution. We also retrieved the two other time-constants, namely $k_{vib}^{HAQ^*} = 3.1$ ps, and $k_{vib}^{DAQ^*} = 4.2$ ps. We compare our retrieved DAQ* signatures to observed dynamics in Figure 14.

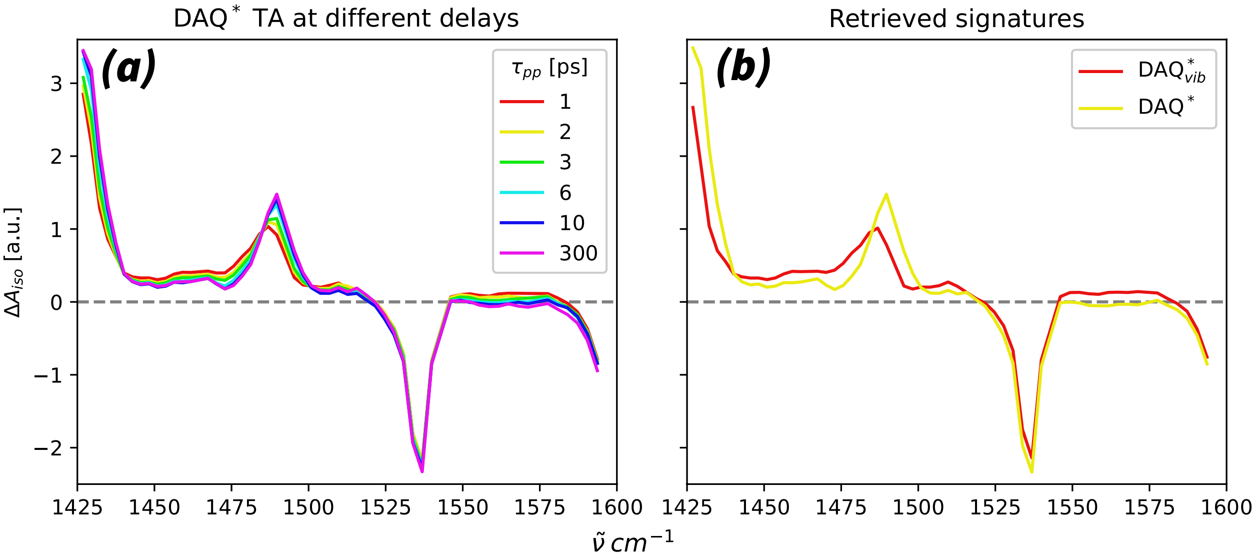

Figure 14: Comparison of transient absorption spectra of DAQ* at different delays (a), with the retrieved signatures using kinetic modelling (b).

Polarisation-resolved kinetic modelling

The method above can be extended to use anisotropy decay to fit polarisation-resolved data. Here, we retrieve signatures for the isotropic data (ΔA_{iso}) together with the polarisation-difference data ($\Delta A_{diff} = \Delta A_{\parallel} - \Delta A_{\perp}$). With a given anisotropy[20] model $R(t)$ for all the transient states, the corresponding populations are

$n_{iso}(t) = n(t)$ and $n_{diff}(t) = n(t) \cdot R(t)$. The quality factor we optimise then is $Q = Q_{iso} + Q_{diff}$.

We note, that this method also allows for the extraction of spectral anisotropy $R(\tilde{\nu}) = \frac{\sigma_{diff}(\tilde{\nu})}{3 \cdot \sigma_{iso}(\tilde{\nu})}$. This can be used to extract angles between transition dipole moments of the $S^0 \to S^1$ electronic transition and ground-state (excited-state) vibrational features at frequencies illustrating strong ground-state bleach (excited-state absorption), using the following formula: $R = \frac{2}{5} \cdot \langle P_2 \left(\overrightarrow{\mu_{S_0 \to S_1}} \cdot \overrightarrow{\mu_{vib}} \right) \rangle$,[20] where $\overrightarrow{\mu}$ represents the transition dipole moment for a given transition and $P_2(x) = \frac{1}{2} \cdot \left(3 \cdot x^2 - 1\right)$ is the second Legendre-polynomial.

We used this fit on our H/DAQ measurements in the first 300 ps, with the additional assumption that the anisotropy decay is governed by a single rate constant. Using this, we obtained that $\left(k_{ani}^{HAQ^*}\right)^{-1} = 60.4$ ps and that $\left(k_{ani}^{DAQ^*}\right)^{-1} = 61.5$ ps. To illustrate the accuracy of this process, we present our DAQ* fits on Figure 15.

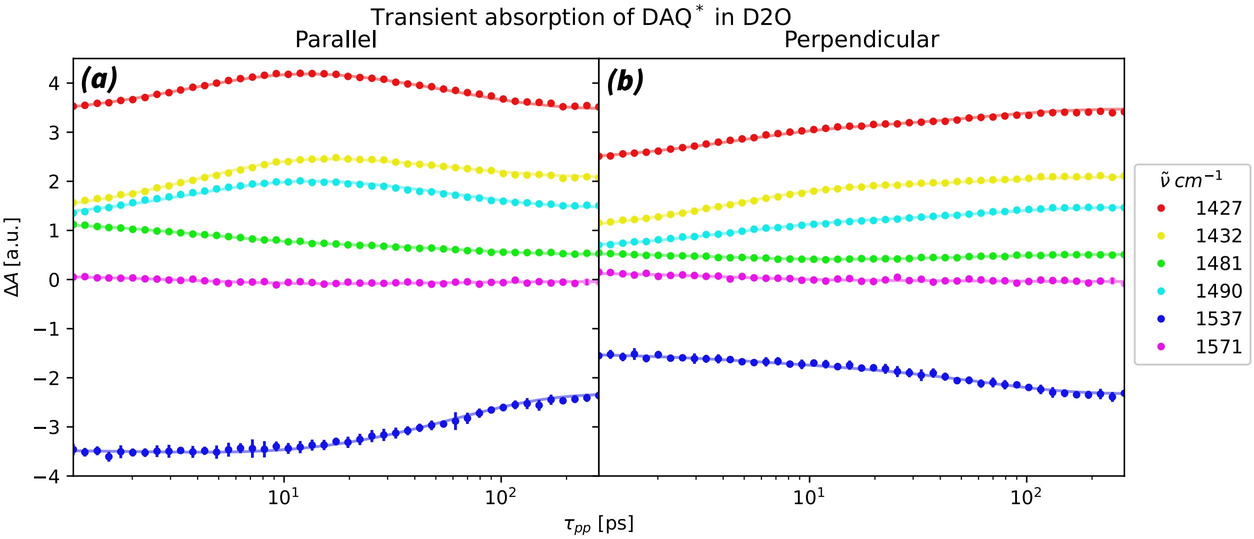

Figure 15: Comparison of transient absorption dynamics of DAQ* at different frequencies in parallel (a), and in perpendicular (b) polarisations. Dots denote experimental data points and lines denote polarisation-resolved kinetic model fits. The error bars denote STD for any given measurement.

Population dynamics using 'soft' kinetic modelling

In soft kinetic modelling, we forgo the assumption that first-order rate constants drive the observable processes. This is the case for ensembles of molecules in different chemical environments, where different sub-populations might have a different reaction rate. This would then cause their population ratio to shift over time, changing the net observable rate constant as well. Because these sub-populations share the same spectral signature, a single first-order rate constant cannot describe their dynamics.

We thus present a mathematical formula for this. In general, we solve the equation system $\frac{\partial}{\partial t}\underline{n} = -\underline{\underline{k}}(t) \cdot \underline{n}$

with the initial conditions that $\underline{n}(t=0)=\underline{n^0}$. We rephrase this equation: $\underline{\underline{k}}(t)=\sum_{ij} k_{ij}(t)\cdot\underline{\underline{M_{ij}}}$ where $\underline{\underline{M_{ij}}}$ is a matrix that is 1 the (i,j) coordinates if i≠j, and 0 otherwise.

The solution is the following: $\underline{n}(t)=e^{\sum_{ij}\{ln(f_{ij}(t))-ln(f_{ij}(t=0))\}\underline{\underline{M_{ij}}}}\cdot\underline{n^0}$, where $\frac{\partial}{\partial t}f_{ij}(t)=-k_{ij}(t)\cdot f_{ij}(t)$. For time-invariant k_{ij}, we get $f_{ij}(t)=exp(-k_{ij}\cdot t)$, which is exactly the solution obtained using the traditional approach.

Owing its flexibility, the main component separating power of this approach does lies with well-defined spectral constraints instead with well-identified dynamics. Thus, we force certain spectral signatures in certain frequency ranges to 0, based on a priori information from FTIR or other TA measurements. We find this approach to be efficient in separating the otherwise fairly correlated H/DAQ* and SI$^-$ signatures.

We also apply this approach to measurement series. Here, the individual measurements are not immediately quantitatively comparable due to slight experimental variations. As such, we optimise for the following global quality factor:

$Q_{global}=\sum_{\tau_{pp},\tilde{\nu},C}(\Delta A_{iso}(\tau_{pp},\tilde{\nu},C)\cdot S_C-\sum_j n^j(\tau_{pp})\cdot\sigma^j(\tilde{\nu}))^2\cdot w^2(\tau_{pp},\tilde{\nu},C)$ where the index C runs through individual measurements and the S_C scaling parameters ensure quantitative comparability. This way we can enforce various initial conditions across all samples, e.g. $n^{AQ^{-*}}(\tau_{pp}=0,c^{H/DSI})=1$.

Applying soft kinetic modelling to analyse H/DSI concentration series

Using the soft modelling approach, we are separate the main components with different signatures, without a priori guessing the kinetic reaction model describing the chemical reaction.

Our generic considerations are the following:

1. We describe AQ^{-*}, H/DAQ* and SI^- signals. We do not account for OH/D^- signals as they have a rather weak and featureless response compared to the molecules above. We estimate that OH/D^- TA accounts only for a few% of the total signal, making its separation unfeasible.

2. We describe the picosecond solvation process of AQ^{-*} using a first-order rate constant k_{solv}.

3. We describe the red-shift of the 1507 cm^{-1} absorption feature with increasing $c^{H/DSI}$. We observed this in TA measurements, and assigned this process to ground-state hydrogen-bonded pair forming as seen in UV-VIS measurements.

4. We describe only singlet excited states, as the proton-uptake reaction from water is rather slow and the excited-state lifetime is typ. $> 10\,ns$, having negligible contribution in the first nanosecond.

Our special considerations are the following:

1. $n^{AQ^{-*}}(\tau_{pp} = 0) = 1$, $n^{H/DAQ^*}(\tau_{pp} = 0) = 0$, $n^{SI^-}(\tau_{pp} = 0) = 0$ for every $c^{H/DSI}$.

2. Based on its FTIR, we enforce $\sigma^{SI^-}(1430\,cm^{-1} < \tilde{\nu} < 1490\,cm^{-1}) = 0$.

3. Based on its AQ^- FTIR and comparison of DAQ FTIR and TA, we enforce $\sigma^{DAQ^*}(1530\,cm^{-1} < \tilde{\nu} < 1570\,cm^{-1}) = 0$ where σ^{DAQ^*} describes the DAQ^* excited-state absorption with AQ^- ground-state bleach as DAQ* was generated via proton transfer.

4. Based on its AQ^- FTIR and comparison of HAQ FTIR and TA, we enforce $\sigma^{HAQ^*}(1555\,cm^{-1} < \tilde{\nu} < 1580\,cm^{-1}) = 0$ where σ^{HAQ^*} describes the HAQ^* excited-state absorption with AQ^- ground-state bleach.

5. We describe the 1507 cm^{-1} ground-state absorption shift of HSI-associated AQ^- using an auxiliary component with the following constraints: $\sigma^{GS\,shift}(\tilde{\nu} = 1507\,cm^{-1}) = 0$, and $n^{GS\,shift} = r^{assoc}(c^{H/DSI}) \cdot (n^{AQ^{-*}} + n^{H/DAQ^*})$ assuming a quick equilibration after excitation.

6. In neat H/D_2O, we observe a minor contribution of H/DAQ at 1535 /1560 cm^{-1}, which varies with c^{AQ^-}. We thus attribute it to the quenching of H/DAQ* by AQ^{-*}. We approximate that this signal accounts for only a few% of the molecules by τ_{pp} =1 ns. Since we do not observe this signal when succinimide is included, we set the fitting weights $w(\tau_{pp} > 200\,ps, \tilde{\nu}^{H/DAQ}, c^{H/DSI} = 0) = 0$.

Our considerations regarding dynamics are the following:

1. In neat water, we use a first-order rate constant to drive the reaction between AQ^{-*} and H/DAQ^{*}.

2. We enforce $k_{PU} : k_{DU}$ based on fluorescence measurements.

3. We use bi and tri-exponential functions (f_{ij}) to generate $k_{P/DU}$ net proton uptake rate governing $AQ^{-*} \rightarrow H/DAQ^{*}$. This way we flexibly account for any possible effects.

4. Similarly, we use separate bi- and tri-exponentials to account for SI^{-} dynamics as well.

5. We enforce the following excited-state lifetime: $k_{ES}^{AQ^{-*}} = k_{fl}^{AQ^{-*}} \cdot \left(\Phi_f^{AQ^{-*}}\right)^{-1}$. We used the calculation that $k_{fl}^{AQ^{-*}} = k_{P/DU} \cdot \left(\frac{k_{fl}^{AQ^{-*}}}{k_{P/DU}}\right)$. We calculated $\left(\frac{k_{fl}^{AQ^{-*}}}{k_{P/DU}}\right)$ and $\Phi_f^{AQ^{-*}}$ based on fluorescence measurements. $\Phi_f^{AQ^{-*}}$ describes what the fluorescence yield of AQ^{-*} would be in abscence of the uptake process.

6. We enforce the following excited-state lifetime: $k_{ES}^{H/DAQ^{*}} = k_{fl}^{H/DAQ^{*}} \cdot \left(\Phi_f^{H/DAQ^{*}}\right)^{-1} \approx k_{P/DU} \cdot \left(\frac{k_{fl}^{AQ^{-*}}}{k_{P/DU}}\right) \cdot \left(\Phi_f^{H/DAQ^{*}}\right)^{-1}$ with the approximation that $k_{fl}^{H/DAQ^{*}} \approx k_{fl}^{AQ^{-*}}$.

To summarize our considerations, we provide a graphical representation in Figure 16.

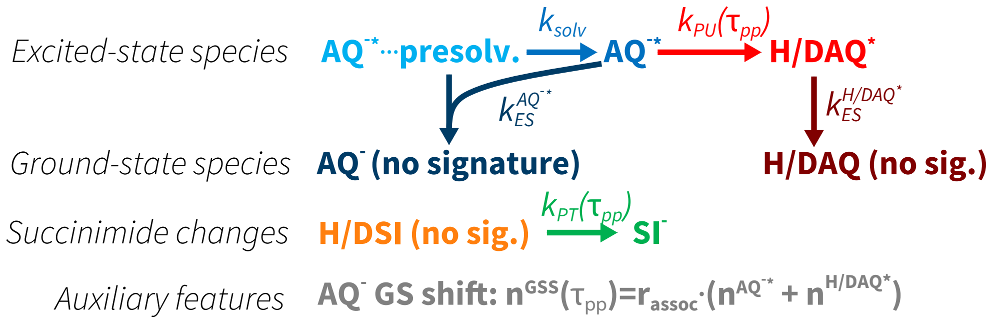

Figure 16: Graphical representation of soft modelling reaction scheme. Different chemical species are illustrated with respective labels. Connecting arrows denote possible reaction pathways, while matching labels describe their rate.

With the conditions set out above, we proceeded to fit the experimental TA data. Because the way we initially set up soft modelling, k_{PU} has a distinguished role in determining a number of rate constants. We therefore established a fitting range for k_{PU} by comparing HAQ^{*} signal at 1565 cm^{-1} in neat H_2O and at 2 M of HSI, at τ_{pp} =1 ns. Because of the saturating trends, we worked with the assumption that $0.5 < n^{HAQ^{*}}(\tau_{pp} = 1\,ns) < 1$, we could calculate that 1.7 ns< $(k_{PU})^{-1}$ <4 ns. To find the best k_{PU}, we thus performed a parametric sweep in this region with a resolution of $\Delta k = (32.5ns)^{-1}$. In each step, we

fixed k_{PU} and used all other parameters to find the best fit on the HSI TA series. This way, we found that $k_{PU}^{best} = 2.46ns$, with the error of $\pm 40\%$ as described before. We illustrate the accuracy of this fit in Figure 17, and the obtained signatures in 18.

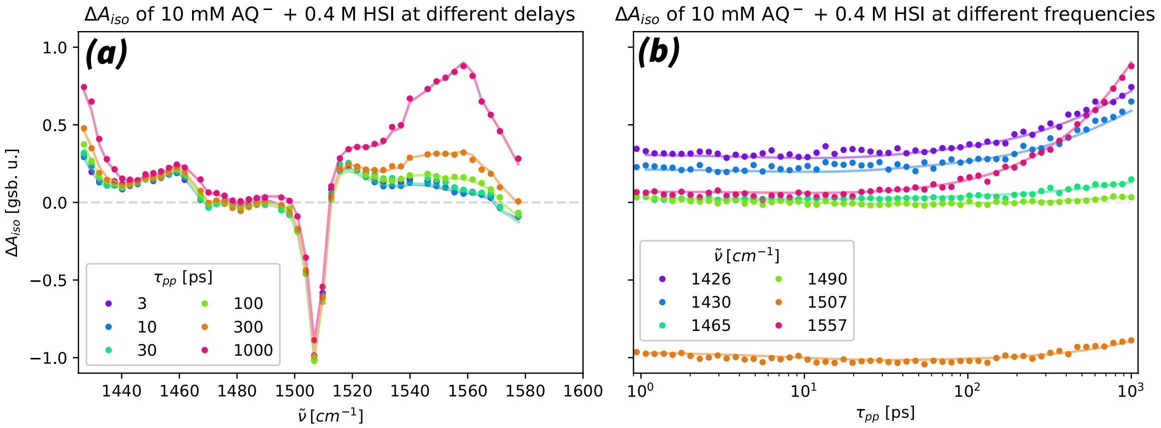

Figure 17: Comparison of transient absorption at different delays (a) and at different frequencies (b) of AQ^{-*} + 0.4 M HSI. Dots denote experimental data points and lines denote reconstructed dataset using soft kinetic modelling.

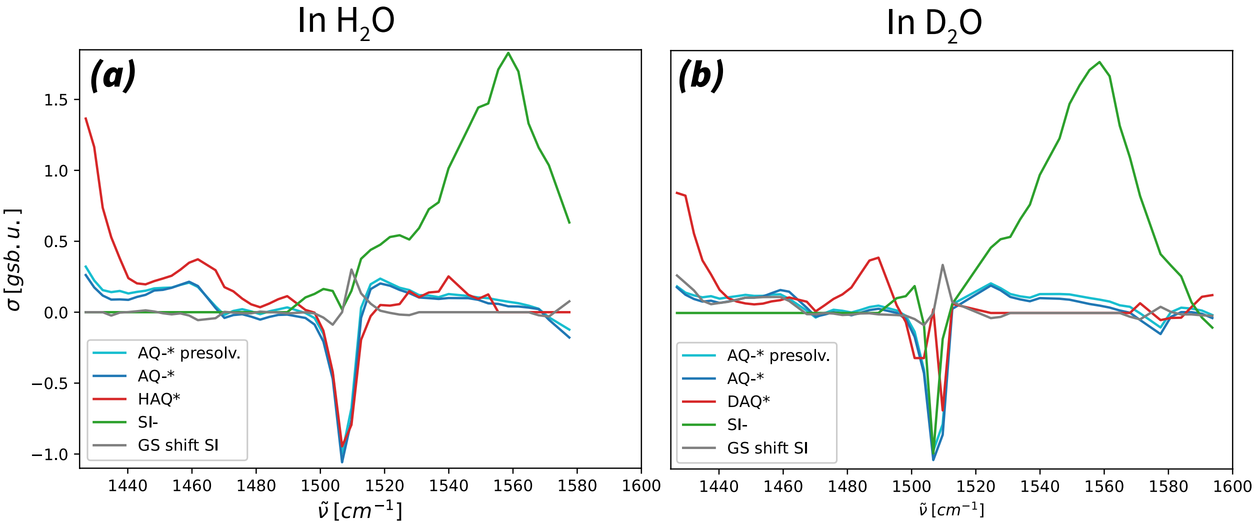

Figure 18: Component signatures resulting from soft kinetic modelling, (a) in H_2O and (b) in D_2O. Presented succinimide signatures are scaled to match each other, and to best match the extended kinetic reaction model.

We note, that the soft-modelling approach leaves a scaling ambiguity between $n^{H/DSI}$ and $\sigma^{H/DSI}$ as the soft model provides no direct connection between $n^{H/DSI}$ and $n^{AQ^{-*}}$. We resolve this in two steps. First, we make $\sigma^{H/DSI}$ is comparable in H_2O and D_2O by scaling then around their maxima. Next, we use the extended kinetic reaction model to find the missing scaling factor. we thus obtain a ratio of $r^{ES} = abs(\Delta A_{iso}^{AQ^-@1507cm^{-1}}) : \Delta A_{iso}^{SI^-@1557cm^{-1}} \approx 1\!:\ 1.8$. If AQ^{-*} has some excited-state signal at 1507 cm^{-1}, then this ratio should be slightly higher than the ratio of the corresponding ground-state absorption signals:

$r^{GS} = \epsilon_{att}^{AQ^{-}@1507cm^{-1}} : \epsilon_{att}^{SI^{-}@1557cm^{-1}}$. We perform a consistency check by directly approximating this ratio using the extinction coefficient ratios in FTIR, using the solvent-subtraction technique we described earlier. After the solvent subtraction, we inversely scale the residual absorption spectra with the relative water amount we subtracted, as well as solute concentration. Using this technique, we obtained $r^{GS} \approx 1 : 1.6$. This falls in line with our expectations of being somewhat smaller than r^{ES}, and the two ratios match within the estimated $\approx 15\%$ error of determining r^{GS}.

Last, we also offer further insights on the 'GS shift' component. This component describes the differential absorption of DSI-associated AQ^{-} species has additional non-zero components at lower frequencies. We hypothesize that is is largely due to HAQ^{*}/DAQ^{*} and HSI/DSI differential absorption as we used non-deuterated HSI to create our D_2O solutions. To avoid this, we will repeat future measurements using pre-deuterated DSI.

Kinetic reaction modelling

Basic reaction model

We combined the obtained spectral signatures and a kinetic reaction model to describe the observed transient absorption dynamics. Here, we define the kinetic equation (see Equation 3) in a similar fashion to the kinetic modelling in the previous section, and illustrate it graphically in Figure 19. We present the obtained best fit constants in Table 6, and the fits themselves on Figure 20.

$$
\begin{aligned}
\frac{\partial}{\partial t}\underline{n} &= \underline{\underline{k}} \cdot \underline{n} \\
\underline{n} &= \begin{bmatrix} n^{AQ^{-*}\cdots H_2O} \\ n^{AQ^{-*}\cdots HSI} \\ n^{HAQ^*} \\ n^{OH^-} \\ n^{SI^-} \end{bmatrix} \\
\underline{\underline{k}} &= \begin{bmatrix} -k_{PU} - k_{ES}^{AQ^{-*}} & 0 & 0 & 0 & 0 \\ 0 & -k_{direct} - k_{ES}^{AQ^{-*}} & 0 & 0 & 0 \\ k_{PU} & k_{direct} & -k_{ES}^{HAQ^*} & 0 & 0 \\ k_{PU} & 0 & 0 & -c^{HSI} \cdot k_{neut} & 0 \\ 0 & k_{direct} & 0 & c^{HSI} \cdot k_{neut} & 0 \end{bmatrix} \\
\underline{n}\left(\tau_{pp} = 0\right) &= \begin{bmatrix} \frac{1 - r^{assoc}}{1 + r^{assoc} \cdot \left(\left(\sigma^{assoc} : \sigma^{free}\right) - 1\right)} \\ \frac{r^{assoc} \cdot \left(\sigma^{assoc} : \sigma^{free}\right)}{1 + r^{assoc} \cdot \left(\left(\sigma^{assoc} : \sigma^{free}\right) - 1\right)} \\ 0 \\ 0 \\ 0 \end{bmatrix}
\end{aligned}
\tag{1}
$$

Table 6: Reaction parameters used our best fit attempt in our basic kinetic reaction model. Relevant absorption cross-sections are measured at excitation energy in pump-probe experiments.

Parameter	In H_2O	In D_2O	Unit
K^{assoc}	0.47	0.51	M^{-1}
σ^{assoc} : σ^{free}	1.58 : 1	1.54 : 1	-
k_{PU}^{-1}	2.46	7.9	ns
k_{neut}^{-1}	35	90	$ps\ M$
k_{direct}^{-1}	300	500	ps

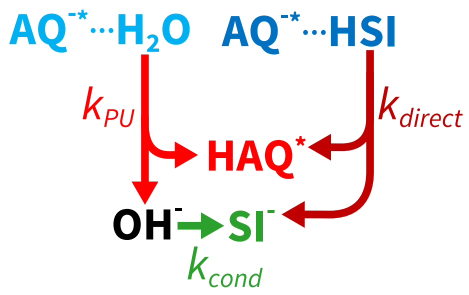

Figure 19: Graphical representation of reaction pathways in our basic reaction model for H_2O. Different chemical species are illustrated with respective labels. Connecting arrows denote possible reaction pathways, while matching labels describe their rate. For clarity of illustration, we omitted excited-state decay pathways.

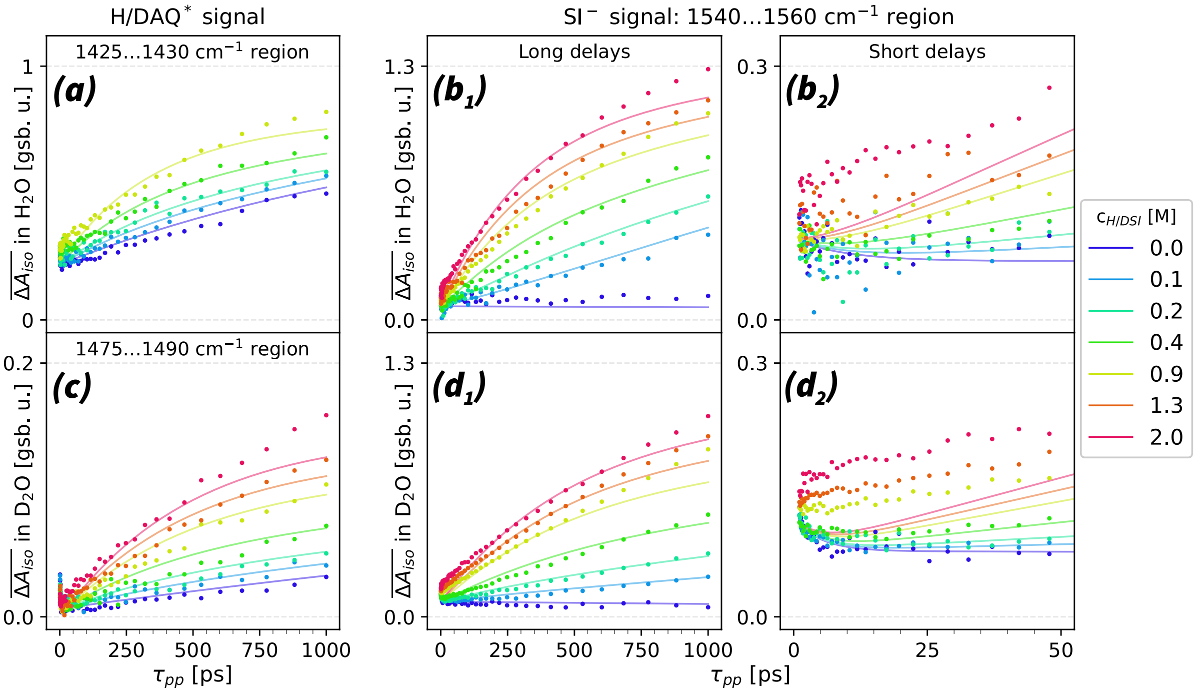

Figure 20: Transient absorption signal as a function of pump-probe delay (τ_{pp}) for different succinimide concentrations ($c^{H/DSI}$).
(a) Spectrally averaged transient absorption dynamics ($\overline{A_{iso}}$) of HAQ* in the 1425-1430 cm^{-1} region for solutions in H_2O. (b_1-b_2) Transient absorption in the 1540-1560 cm^{-1} spectral region in H_2O. (c) Transient absorption of DAQ* in the 1475-1490 cm^{-1} region, for solutions in D_2O. (d_1-d_2) Transient absorption in the 1540-1560 cm^{-1} spectral region in D_2O.
The presented experimental plots are normalised to an identical initial AQ^{-*} population. The solid lines represent fits to the data using the basic kinetic reaction model described in the SI.

We note, that solvent relaxation is not explicitly included in the basic or the extended model, and neither is the ground-state shift of the hydrogen-bonded species. To use their signatures, we connect the two modelling schemes with the following equations:

$$\begin{aligned}
n_{presolv,soft}^{AQ^{-*}} &= \left(n_{kin.react.}^{AQ^{-*}\cdots H_2O} + n_{kin.react.}^{AQ^{-*}\cdots HSI}\right) \cdot exp(-\tau_{pp} \cdot k_{solv}) \\
n_{soft}^{AQ^{-*}} &= \left(n_{kin.react.}^{AQ^{-*}\cdots H_2O} + n_{kin.react.}^{AQ^{-*}\cdots HSI}\right) \cdot (1 - exp(-\tau_{pp} \cdot k_{solv})) \\
n_{soft}^{GSshift} &= r^{assoc}(c^{HSI}) \cdot \left(n_{kin.react.}^{AQ^{-*}\cdots H_2O} + n_{kin.react.}^{AQ^{-*}\cdots HSI} + n_{kin.react.}^{HAQ^{*}}\right)
\end{aligned} \tag{2}$$

Extended model

We also offer an extended kinetic reaction model including two additional phenomena.

$$\frac{\partial}{\partial t}\underline{n} = \underline{\underline{k}} \cdot \underline{n}$$

$$\underline{n} = \begin{bmatrix} n^{AQ^{-*}\cdots H_2O} \\ n^{AQ^{-*}\cdots HSI} \\ n^{AQ^{-*}(HSI)_2} \\ n^{HAQ^*} \\ n^{OH^-} \\ n^{SI^-} \end{bmatrix}$$

$$\underline{\underline{k}} = \begin{bmatrix} -k_{PU} - k_{dissoc} - k_{ES}^{AQ^{-*}} & +k^{assoc} \cdot c^{HSI} & 0 & 0 & 0 & 0 \\ k_{dissoc} & -k_{direct} - k_{ES}^{AQ^{-*}} - k^{assoc} \cdot c^{HSI} & 0 & 0 & 0 & 0 \\ 0 & 0 & -k_{trio} & 0 & 0 & 0 \\ k_{PU} & k_{direct} & k_{trio} & -k_{ES}^{HAQ^*} & 0 & 0 \\ k_{PU} & 0 & 0 & 0 & -c^{HSI} \cdot k_{neut} & 0 \\ 0 & k_{direct} & k_{trio} & 0 & c^{HSI} \cdot k_{neut} & 0 \end{bmatrix}$$

$$\underline{n}\left(\tau_{pp} = 0\right) = \begin{bmatrix} \frac{1 - r^{assoc}}{1 + r^{assoc} \cdot \left(\left(\sigma^{assoc} : \sigma^{free}\right) - 1\right)} \\ \frac{r^{assoc} \cdot \left(\sigma^{assoc} : \sigma^{free}\right)}{1 + r^{assoc} \cdot \left(\left(\sigma^{assoc} : \sigma^{free}\right) - 1\right)} \cdot \left(1 - r^{trio}\right) \\ \frac{r^{assoc} \cdot \left(\sigma^{assoc} : \sigma^{free}\right)}{1 + r^{assoc} \cdot \left(\left(\sigma^{assoc} : \sigma^{free}\right) - 1\right)} \cdot r^{trio} \\ 0 \\ 0 \\ 0 \end{bmatrix} \tag{3}$$

Last, we assess the whether the uncertainty of knowing k_{PU} impacts the our proposed model. We thus we repeated the entire fitting procedure for the HSI TA series using $k_{PU} = (1.89 \text{ ns})^{-1}$ and $k_{PU} = (3.53 \text{ ns})^{-1}$, which values are approximately at the edges of the confidence interval for k_{PU}. We found that it is possible to adjust the k_{direct} and k_{neut} so that the fits remain similarly accurate. We summarize the fit results in Table 7.

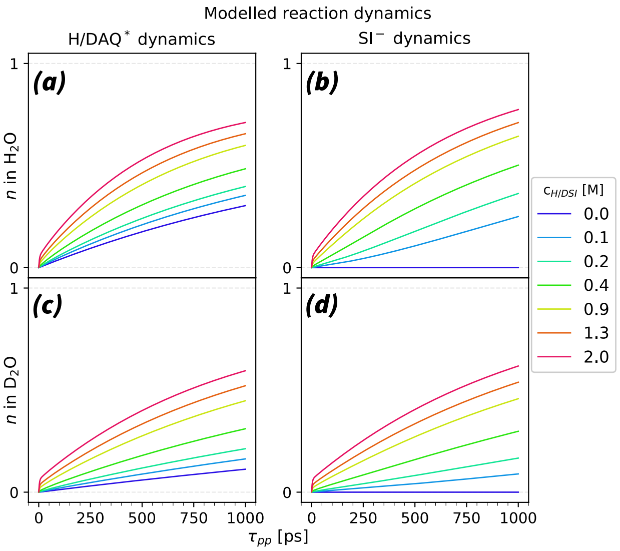

Figure 21: Population dynamics derived from the presented kinetic reaction model. (a) HAQ* in H_2O. (b) SI^- in H_2O. (c) DAQ* in D_2O. (d) SI^- in D_2O.

Table 7: Alternative fit parameters for the reaction kinetics in H_2O. Relative uncertainties are calculated from parameter changes in fits with different k_{PU}-s.

Parameter	Slowest k_{PU}	Mean k_{PU}	Fastest k_{PU}	Rel. uncertainty	Unit
k_{PU}^{-1}	3.53	2.46	1.89	$\pm 40\%$	*ns*
k_{neut}^{-1}	42	50	62	$\mp 23\%$	*ps M*
k_{direct}^{-1}	460	360	330	$\pm 22\%$	*ps*
$abs(\Delta A_{iso}^{AQ^-@1507cm^{-1}}) : \Delta A_{iso}^{SI^-@1557cm^{-1}}$	1:2.1	1:1.8	1:1.7	$\pm 14\%$	-

References

(1) Nawara, K.; Waluk, J. Goodbye to Quinine in Sulfuric Acid Solutions as a Fluorescence Quantum Yield Standard. *Analytical Chemistry* **2019**, *91*, 5389–5394, PMID: 30907575.

(2) Nawara, K.; Waluk, J. Fluorescence quantum yield determination using simultaneous double-beam absorption measurement. *Measurement* **2020**, *165*, 108159.

(3) Würth, C.; Grabolle, M.; Pauli, J.; Spieles, M.; Resch-Genger, U. Comparison of methods and achievable uncertainties for the relative and absolute measurement of photoluminescence quantum yields. *Analytical chemistry* **2011**, *83*, 3431–3439.

(4) Optics Handling and Care Tutorial. `https://www.thorlabs.com/newgrouppage9.cfm?objectgroup_id=9025`.

(5) MacDonald, B. C.; Lvin, S. J.; Patterson, H. Correction of fluorescence inner filter effects and the partitioning of pyrene to dissolved organic carbon. *Analytica chimica acta* **1997**, *338*, 155–162.

(6) Brouwer, A. M. Standards for photoluminescence quantum yield measurements in solution (IUPAC Technical Report). *Pure and Applied Chemistry* **2011**, *83*, 2213–2228.

(7) Resch-Genger, U.; Rurack, K. Determination of the photoluminescence quantum yield of dilute dye solutions (IUPAC Technical Report). *Pure & Applied Chemistry* **2013**, *85*.

(8) Mehata, M.; Tripathi, H. Fluorescence quenching of 6-methoxyquinoline: an indicator for sensing chloride ion in aqueous media. *Journal of luminescence* **2002**, *99*, 47–52.

(9) Siano, D. B. The log-normal distribution function. *Journal of Chemical Education* **1972**, *49*, 755.

(10) Driscoll, E. W.; Hunt, J. R.; Dawlaty, J. M. Photobasicity in Quinolines: Origin and Tunability via the Substituents' Hammett Parameters. *The Journal of Physical Chemistry Letters* **2016**, *7*, 2093–2099, PMID: 27195691.

(11) Venyaminov, S. Y.; Prendergast, F. G. Water (H2O and D2O) molar absorptivity in the 1000-4000 cm^{-1} range and quantitative infrared spectroscopy of aqueous solutions. *Analytical biochemistry* **1997**, *248*, 234–245.

(12) National Bureau of Standards, *Tables of Molecular Vibrational Frequencies*; U.S. Dep. of Commerce, 1967.

(13) Mohrig, J. R.; Hammond, C. N.; Schatz, P. F. *Techniques in Organic Chemistry*; Freeman, 2006.

(14) Kristiansson, O.; Lindgren, J.; De Villepin, J. A quantitative infrared spectroscopic method for the study of the hydration of ions in aqueous solutions. *The Journal of Physical Chemistry* **1988**, *92*, 2680–2685.

(15) Stamboliyska, B.; Binev, Y. I.; Radomirska, V.; Tsenov, J.; Juchnovski, I. IR spectra and structure of 2, 5-pyrrolidinedione (succinimide) and of its nitranion: experimental and ab initio MO studies. *Journal of Molecular Structure* **2000**, *516*, 237–245.

(16) Khan, M. N.; Khan, A. A. Kinetics and mechanism of hydrolysis of succinimide in highly alkaline medium. *The Journal of Organic Chemistry* **1975**, *40*, 1793–1794.

(17) Hamm, P.; Wiemann, S.; Zurek, M.; Zinth, W. Highly sensitive multichannel spectrometer for subpicosecond spectroscopy in the midinfrared. *Optics letters* **1994**, *19*, 1642–1644.

(18) Agrawal, G. P.; Baldeck, P.; Alfano, R. Temporal and spectral effects of cross-phase modulation on copropagating ultrashort pulses in optical fibers. *Physical Review A* **1989**, *40*, 5063.

(19) Mohammed, O. F.; Dreyer, J.; Magnes, B.-Z.; Pines, E.; Nibbering, E. T. Solvent-dependent photoacidity state of pyranine monitored by transient mid-infrared spectroscopy. *ChemPhysChem* **2005**, *6*, 625–636.

(20) Lipari, G.; Szabo, A. Effect of librational motion on fluorescence depolarization and nuclear magnetic resonance relaxation in macromolecules and membranes. *Biophysical journal* **1980**, *30*, 489–506.
